# HLA-DR cancer cells expression correlates with T cell infiltration and is enriched in lung adenocarcinoma with indolent behavior

**DOI:** 10.1038/s41598-021-93807-3

**Published:** 2021-07-13

**Authors:** Maria-Fernanda Senosain, Yong Zou, Tatiana Novitskaya, Georgii Vasiukov, Aneri B. Balar, Dianna J. Rowe, Deon B. Doxie, Jonathan M. Lehman, Rosana Eisenberg, Fabien Maldonado, Andries Zijlstra, Sergey V. Novitskiy, Jonathan M. Irish, Pierre P. Massion

**Affiliations:** 1 Cancer Biology Graduate Program, Vanderbilt University, Nashville, TN USA; 2 Division of Allergy, Pulmonary, and Critical Care Medicine, Department of Medicine, Vanderbilt University Medical Center, Nashville, TN USA; 3 Cancer Early Detection and Prevention Initiative, Vanderbilt-Ingram Cancer Center, Vanderbilt University Medical Center, Nashville, TN USA; 4 Vanderbilt-Ingram Cancer Center, Vanderbilt University Medical Center, Nashville, TN USA; 5 Department of Cell and Developmental Biology, Vanderbilt University, Nashville, TN USA; 6 Division of Hematology/Oncology, Department of Medicine, Vanderbilt University Medical Center, Nashville, TN USA; 7 Department of Pathology, Microbiology and Immunology, Vanderbilt University Medical Center, Nashville, TN USA; 8 US Department of Veterans Affairs, Tennessee Valley Healthcare System, Nashville, TN USA

**Keywords:** Cancer, Immunology, Oncology

## Abstract

Lung adenocarcinoma (ADC) is a heterogeneous group of tumors associated with different survival rates, even when detected at an early stage. Here, we aim to investigate whether CyTOF identifies cellular and molecular predictors of tumor behavior. We developed and validated a CyTOF panel of 34 antibodies in four ADC cell lines and PBMC. We tested our panel in a set of 10 ADCs, classified into long- (LPS) (n = 4) and short-predicted survival (SPS) (n = 6) based on radiomics features. We identified cellular subpopulations of epithelial cancer cells (ECC) and their microenvironment and validated our results by multiplex immunofluorescence (mIF) applied to a tissue microarray (TMA) of LPS and SPS ADCs. The antibody panel captured the phenotypical differences in ADC cell lines and PBMC. LPS ADCs had a higher proportion of immune cells. ECC clusters (ECCc) were identified and uncovered two ADC groups. ECCc with high HLA-DR expression were correlated with CD4+ and CD8+ T cells, with LPS samples being enriched for those clusters. We confirmed a positive correlation between HLA-DR expression on ECC and T cell number by mIF staining on TMA slides. Spatial analysis demonstrated shorter distances from T cells to the nearest ECC in LPS. Our results demonstrate a distinctive cellular profile of ECC and their microenvironment in ADC. We showed that HLA-DR expression in ECC is correlated with T cell infiltration, and that a set of ADCs with high abundance of HLA-DR+ ECCc and T cells is enriched in LPS samples. This suggests new insights into the role of antigen presenting tumor cells in tumorigenesis.

## Introduction

Recently, the National Lung Screening Trial (NLST) reported a 20% relative mortality risk reduction using low-dose computed tomography (CT) over chest X-ray screening^1^. However, lung tumors detected through CT screening range from indolent to aggressive. Aggressive lung cancers have doubling times of 50 to 150 days, yet CT screening has been shown to detect slow growing tumors with doubling times of 400 days or more^2^. Lung cancer screening bears the inherent risk of overdiagnosis in up to 18% of tumors^3^. Recent efforts in radiomics have been reported to predict this phenomenon, however its biological determinants remain unknown^4–6^.

Lung adenocarcinoma (ADC) is a highly heterogeneous disease. Assuming that subpopulations may be responsible for a particular behavior, these may be rare and difficult to detect at an early stage with standard bulk analyses^7–9^. Until recently, the molecular profiling of tumors has been based on an average phenotype of hundreds of thousands of cells, including neoplastic cells and cells of the tumor microenvironment (TME). Although this approach has proven to be useful in many applications, there is a significant loss of information, particularly affecting the detection of rare cell subsets that could be responsible for cancer initiation, plasticity and recurrence. Emerging single-cell technologies can overcome such limitation, providing high resolution information essential for a better understanding of the tumor cellular composition^10^. Among those, mass cytometry is a rapidly evolving technology capable of measuring the expression of $\sim$ 40 proteins on individual cells using antibodies labeled with heavy metal isotopes^11^. To date, some studies have investigated ADC from a single-cell perspective^12–17^, however the molecular determinants of early ADC behavior as for why some tumors progress faster than others remain unknown.

Here, we hypothesized that single-cell proteomic analysis of early stage adenocarcinoma of the lung will provide new insights into the cellular and molecular determinants of indolent and aggressive tumors which in turn may offer novel and personalized avenues for intervention. We developed a comprehensive mass cytometry antibody panel that will allow us to investigate ADC behavior, which includes markers for cellular lineage, tumor cell markers and signaling pathways. To this end, we have validated our panel using ADC cell lines and PBMC and we present the analysis of a set of ten early stage primary ADCs of the lung with indolent and aggressive behaviors showing some valuable insights on immunogenicity of the tumors.

## Results

### ADC mass cytometry antibody panel captures the cellular diversity between ADC cell lines and PBMC

**Figure 1:**
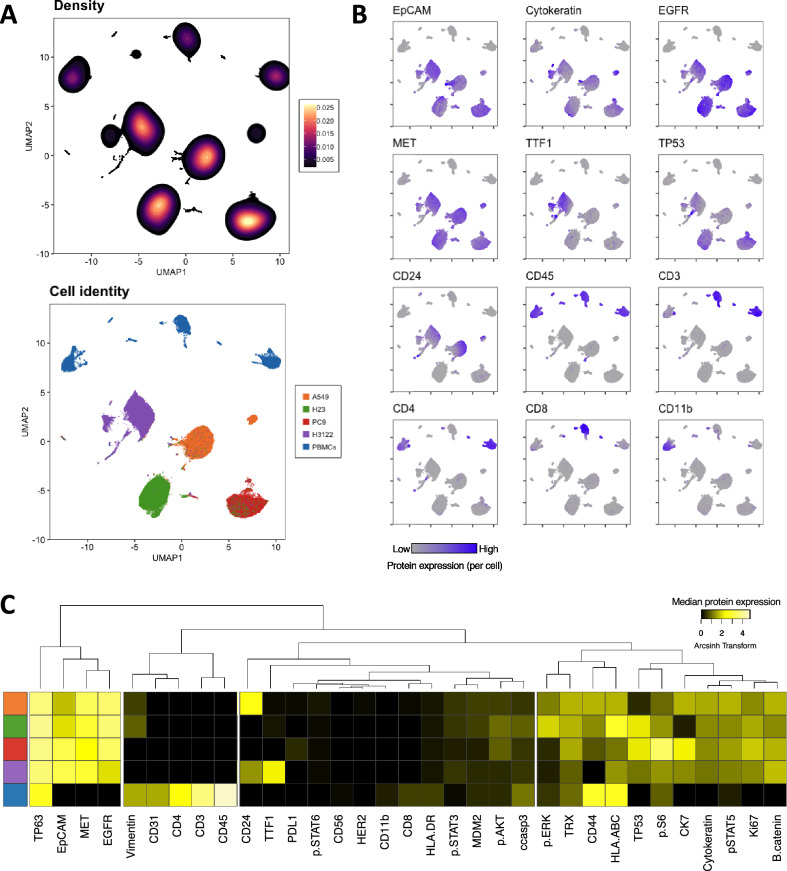
Mass cytometry panel and unsupervised computational analysis capture cellular diversity in ADC cell lines and PBMC. (**A**) Density (above) and cell identity (below) UMAP representations show separation of the cellular populations based on single-cell protein expression. (**B**) UMAP plots correspond to the same cells from (**A**) showing single cell expression of the labeled protein. (**C**) Heatmap shows median protein expression of arcsinh transformed values (cofactor = 5) for each protein on each cell population. Colors on the left represent the cellular populations and match those represented in (**A**).

To validate our mass cytometry panel, we used a combination of ADC cell lines that harbor different mutations and therefore have different protein expression patterns (Table E1). We also included PBMC from a healthy donor in the mix to mimic the immune cells that can be found in a tumor. All cells were pooled in the same proportion, stained and run through the CyTOF machine as a single sample. Additionally, cells were run separately to confirm our findings. Protein expression by cell line was consistent across replicates (Figs. E1–E7). Dimensionality reduction algorithm UMAP^18^ allowed us to visualize the multiple parameters measured in a two dimensional map (Fig. 1A,B). Our panel captured phenotypic differences among the cell lines and PBMC in the parameter space, visualized as independent islands in the UMAP plot (Fig. 1A). Epithelial markers EpCAM, pan-cytokeratin and cytokeratin 7 were positive in ADC cell lines, but not always expressed on the same cells (Fig. 1B). Receptor tyrosine kinases EGFR and MET were highly expressed in all ADC cell lines as expected. Cell line H3122 was positive for TTF1 as previously reported^19^, and cell lines PC9 and H23 which harbor inactivating TP53 mutations expressed high levels of the latter (Table E1). A549 expressed high levels of CD24. Human PBMC were all CD45 positive and divided into three major islands: CD3+ CD4+ (T helper cells), CD3+ CD8+ (cytotoxic T cells), and CD3− CD11b+ cells (myeloid cells). Additionally, basal kinase activity as represented by phosphorylation of ERK, S6, STAT5 and, in lesser degree, AKT was detected mostly in ADC cell lines, reflecting the constitutive activation of these pathways (Fig. 1C).

**Figure 2:**
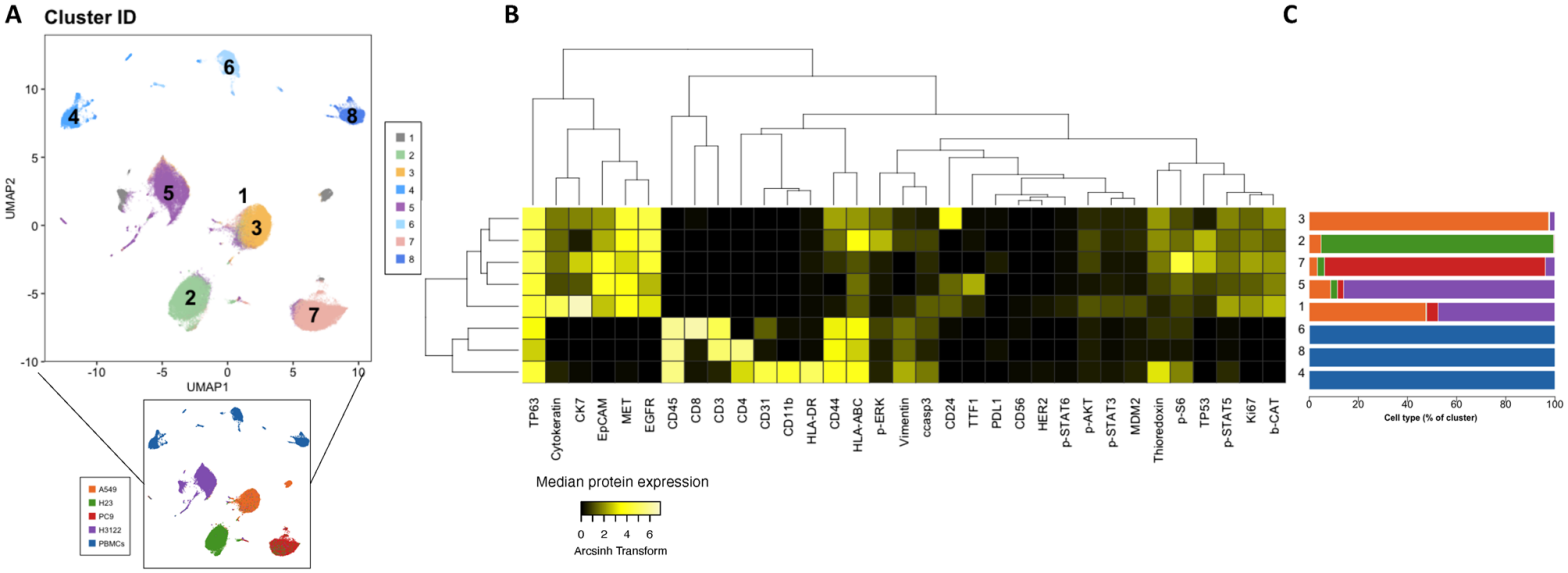
Clustering analysis of ADC cell lines and PBMC. (**A**) UMAP plot is the same as in Fig. 1 but colors represent 8 clusters obtained with k-means. (**B**) Heatmap shows median protein expression of arcsinh transformed values (cofactor = 5) for each protein on each cluster. (**C**) Stacked barplots represent cluster composition (percentage per cell type). Colors match those represented in (**A**) (bottom).

To test if our clustering strategy was successful in identifying the different cell types in the mix, we determined the optimal number of clusters and studied their composition. To determine the optimal number of clusters *k* to target with k-means clustering, we used the ‘elbow’ criterion, for which the total within-cluster sum of squares was calculated for a range of values of *k*^20^. Clustering was performed with *k* = 8. The resulting clusters represented with high accuracy the different cell types present in the mix (Fig. 2). Cluster 2 was 94.6% composed by H23 cells, cluster 3 was 97.4% composed by A549 cells; cluster 5 was 86% composed by H3122 cells and cluster 7 was 90% composed of PC9 cells. For the immune clusters, clusters 4, 6 and 8 were 100% composed by PBMC. Based on their protein expression, these could be annotated as CD11b+ monocytes, CD8+ T cells and CD4+ T cells, respectively. Finally, cluster 1 is a mix of cells dominated by A549 and H3122 cells, driven by a high pan-cytokeratin and cytokeratin 7 expression. Altogether, these results show that our mass cytometry antibody panel can successfully identify different cancer subsets as well as some immune populations.

### Mass cytometry analysis identifies main cell types in ADCs and captures differences between tumors with long and short predicted survival

**Figure 3:**
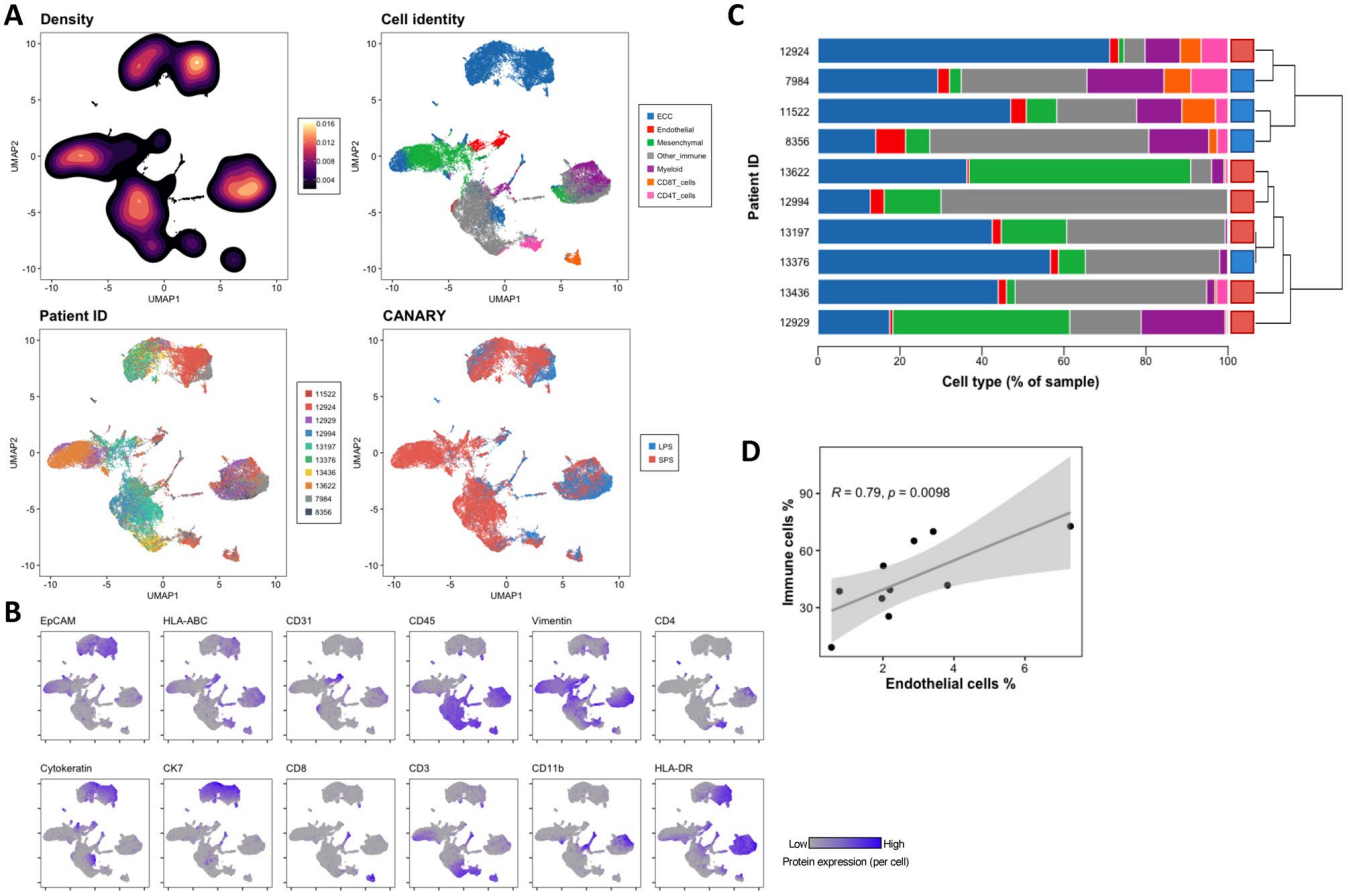
Mass cytometry antibody panel distinguishes epithelial and non-epithelial cell types in 10 early ADCs. (**A**) UMAP plots of a random sample of 4000 cells per patient colored by Density, Cell identity, Patient ID and CANARY prediction. Seven cell types were identified based on k-means clustering and marker expression profiles. Patient CANARY risk stratification is represented as a light blue for long-predicted survival (LPS) and dark blue for and short-predicted survival (SPS). (**B**) UMAP plots correspond to the same cells from (**A**) showing single cell expression of selected labeled protein. (**C**) Stacked barplots with cell type percentage per patient. Colors match those in (**A**) Cell identity plot. Dendrogram was calculated from a patient-patient Spearman correlation matrix. (**D**) Spearman correlation analysis of the relative abundance of immune cells vs. endothelial cells.

Lung ADCs human samples characterized by different predicted behavior classified into long- (LPS) (n = 4) and short-predicted survival (SPS) (n = 6) were stained with our antibody panel (Table 1, Fig. E8). We identified the major cell types (ECC, endothelial, mesenchymal and immune cells) based on the expression of protein markers (Fig. 3B). EpCAM+/pan-cytokeratin+/cytokeratin 7+ cells were annotated as ECC; CD31+/CD45− cells were annotated as endothelial cells; vimentin+ /CD31−/ CD45− and negative for epithelial markers cells were annotated as mesenchymal cells. All CD45+ cells and negative for epithelial markers were annotated as immune cells. The latter were further classified into T helper cells (CD3+/CD4+/CD8−), cytotoxic T cells (CD3+/CD8+/CD4−), myeloid cells (CD11b+/CD3−) and the remainder CD45+ cells were annotated as “Other immune”. While the number of cells acquired varied between samples, we included all cells collected for each tumor in the analysis and used the cell type relative abundances (i.e. percentages) for comparisons.

**Table 1:** Mass cytometry antibody panel for lung adenocarcinoma.

Antigen	Isotope	Level	Clone	Source	Catalog #
EpCAM	141-Pr	Surface	9C4	Fluidigm	3141006B
c-caspase3	142-Nd	Intracellular	D3E9	Fluidigm	3142004A
TP53^a^	143-Nd	Intracellular	DO-7	Biolegend	645802
HLA-ABC	144-Nd	Surface	W6/32	Fluidigm	3144017B
CD31	145-Nd	Surface	WM59	Fluidigm	3145004B
Thioredoxin	146-Nd	Intracellular	2G11/TRX	Fluidigm	3146016B
b-CAT	147-Sm	Intracellular	D10A8	Fluidigm	3147005A
HER2	148Nd	Surface	29D8	Fluidigm	3148011A
p-STAT6	149-Sm	Intracellular	18/P-Stat6	Fluidigm	3149004A
p-STAT5	150-Nd	Intracellular	Y694	Fluidigm	3150005A
TTF1^a^	151-Eu	Intracellular	D2E8	CST	12373
p-AKT	152-Sm	Intracellular	D9E	Fluidigm	3152005A
ki67^a^	153-Eu	Intracellular	ki67	Biolegend	350523
CD45	154-Sm	Surface	HI30	Fluidigm	3154001B
CD56/NCAM	155-Gd	Surface	B159	Fluidigm	3155008B
Vimentin	156-Gd	Intracellular	RV202	Fluidigm	3156023A
p-STAT3	158-Gd	Intracellular	Y705	Fluidigm	3158005A
CD4^a^	159-Tb	Surface	RPA T4	Biolegend	300502
MDM2^a^	160-Gd	Intracellular	Polyclonal	Abcam	ab38618
Cytokeratin^a^	161-Dy	Intracellular	C-11	Abcam	ab7753
MET^a^	162-Dy	Surface	L6E7	CST	8741
TP63^a^	163-Dy	Intracellular	W15093A	Biolegend	687202
CK7	164-Dy	Intracellular	RCK105	Fluidigm	3164020A
EGFR^a^	165-Ho	Surface	AY13	Biolegend	352902
CD44	166-Er	Surface	BJ18	Fluidigm	3166001B
p-ERK	167-Er	Intracellular	D13.14.4E	Fluidigm	3167005A
CD8	168-Er	Surface	RPA-T8	Fluidigm	3168002B
CD24	169-Tm	Surface	ML5	Fluidigm	3169004B
CD3e	170-Yb	Surface	SP34-2	Fluidigm	3170007B
CD11b^a^	171-Yb	Surface	ICRF44	Biolegend	301337
p-S6	172-Yb	Intracellular	N7-548	Fluidigm	3172008A
HLA-DR	174-Yb	Surface	L243	Fluidigm	3172008A
CD274/PDL1	175-Lu	Surface	29E.2A3	Fluidigm	3175017B
Histone H3	176-Yb	Intracellular	D1H2	Fluidigm	3176016A

^a^Customized conjugated antibodies.

Figure 3A is a representation of an equal sampling of annotated cell types of the 10 tumors using dimensionality reduction algorithm UMAP^18^. Cell types separated based on their marker expression (Fig. 3A, Cell identity). Additionally, events (i.e. cells) did not cluster by sample but were mixed among the different islands in the plot (Fig. 3A, Patient ID). We further investigated the distribution of these cell types across the 10 tumors by performing hierarchical clustering on the correlation matrix based on the subpopulations relative abundances (Fig. 3C). Samples clustered in two main groups, one enriched in T cells and myeloid cells and one with lower to no abundance of those cell types and higher abundance of mesenchymal cells on average. The first group of samples was composed by 3 LPS samples (7984, 11522, 8356) and one SPS sample (12924). The other group of samples was mainly composed of SPS samples (13622, 12994, 13197, 13436, 12929) and one LPS sample (13376) (Table E4). Additionally, we found a statistically significant positive correlation between endothelial cells and immune cells in the ADC samples (Figs. 3D, E9). When LPS and SPS tumor samples were compared, we found that LPS had a higher median percentage of endothelial cells and immune subtypes, whereas SPS samples had a higher median percentage of fibroblasts/mesenchymal cells (Fig. E10).

We compared LPS vs SPS protein expression by cell types (Figs. E10–E16). We found a tendency towards a higher expression of HLA-DR and HLA-ABC in endothelial cells from LPS tumors (Fig. E11). In epithelial and mesenchymal cells there was higher HLA-DR expression in LPS compared to SPS tumors, with the latter cell type showing a significant difference (p = 0.038) (Figs. E12, 13). The immune cells as a whole also showed a tendency towards higher HLA-DR expression in LPS tumors (Fig. E14). CD8+ T cells showed a significantly higher expression of HLA-ABC in LPS tumors (p = 0.032) (Fig. E15). CD4+ T cells showed a tendency towards higher expression of activation marker CD44 in LPS tumors (Fig. E16). Finally, myeloid cells presented a tendency towards higher expression of HLA-ABC and HLA-DR in LPS tumors (Fig. E17). To confirm that the HLA-DR higher expression in most cell types of LPS tumors was not due to an artifact of the antibody, we assessed the expression of this protein in our batch control cell lines A549 and Ramos (Fig. E18). Results were consistent across batches, with A549 showing minimal expression of HLA-DR and Ramos showing high expression of the protein in question as expected.

Based on these results, we conclude that our mass cytometry antibody panel enables the identification of major cell types in ADCs, allowing for comparison across tumors of different predicted behavior. We found that our set of samples divided in two main groups based on their cellular composition, one enriched on T cells (LPS predominant) and one depleted on T cells (SPS predominant). Additionally, we found a tendency towards a higher HLA-DR expression in LPS samples, suggesting an immunogenic profile on these tumors.

### Unsupervised analysis of ECC reveals HLA-DR+ subsets associated with T cell infiltration

**Figure 4:**
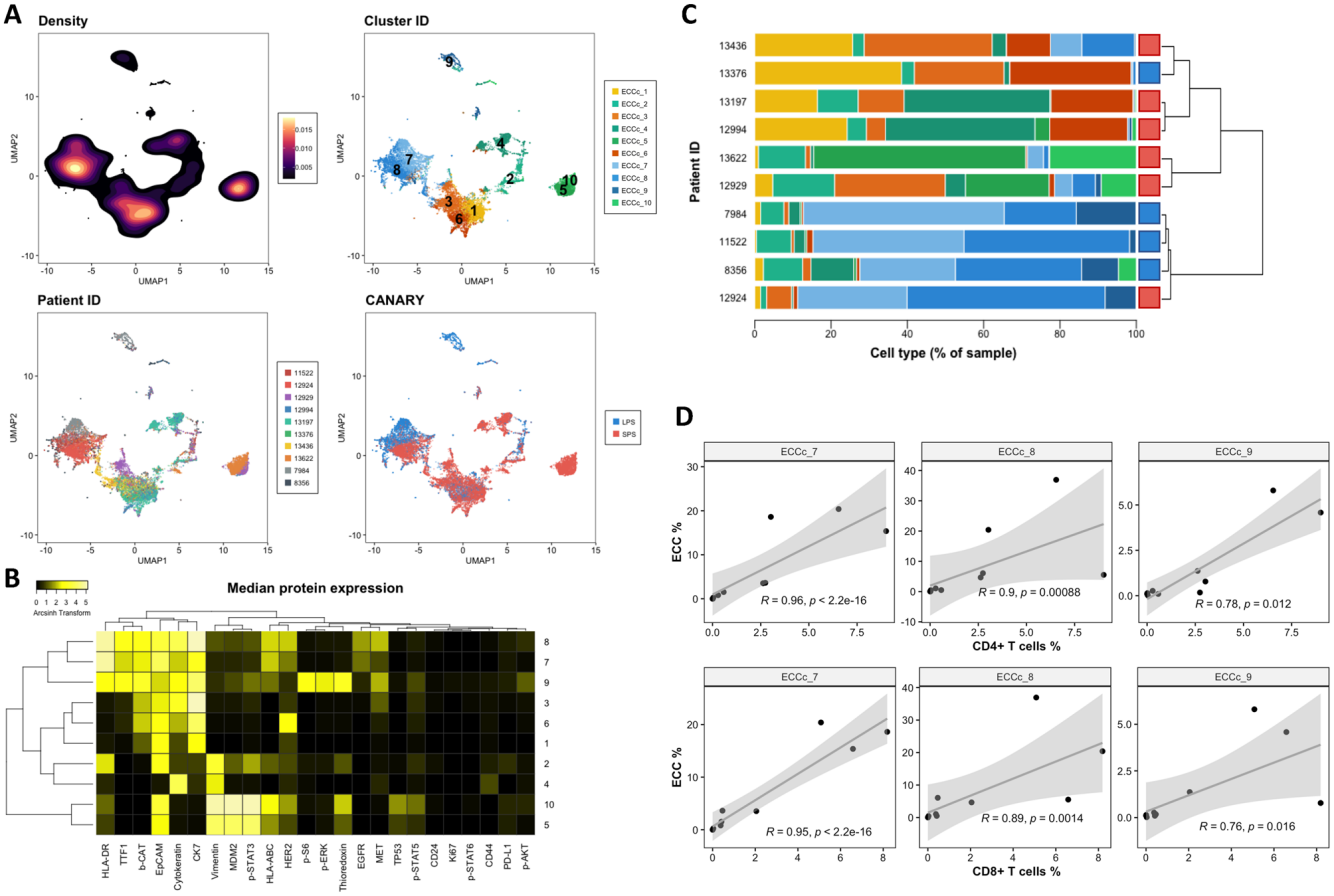
Unsupervised analysis of ECC reveals intra- and inter-tumor heterogeneity. (**A**) UMAP plots of a random sample of 2000 ECC per patient colored by Density, Cell identity, Patient ID and CANARY prediction. Ten clusters were obtained based on k-means clustering. Patient CANARY risk stratification is represented as a light blue for long-predicted survival (LPS) and dark blue for and short-predicted survival (SPS). (**B**) Heatmap shows median protein expression of arcsinh transformed values (cofactor = 5) for each protein on each ECCc. (**C**) Stacked barplots with ECCc percentage per patient. Colors match those in (**A**). Dendrogram was calculated from a patient-patient Spearman correlation matrix. (**D**) Spearman correlation analysis of the relative abundance of ECCc 7, 8 and 9 vs CD4+ and CD8+ T cells, respectively.

Because distinct subpopulations of malignant cells have been associated with disease outcome^9^, we tested whether our antibody panel detects different subsets of ECC and whether LPS or SPS tumors are particularly enriched for any subset. We computationally extracted the ECC of each tumor from the pool of cells (Fig. 3). We used *k* = 10 to achieve more granularity and dig deeper into the differences of the ECC. Figure 4A is an equal-sampling representation of the 10 ECCc of the 10 ADC samples using dimensionality reduction algorithm UMAP^18^. ECCc separated based on their protein expression (Fig. 4A,B, Cluster ID) and cells did not grouped by sample but were mixed among the different islands in the plot (Fig. 4A, Patient ID). We then assessed the sample ECCc composition across the 10 tumors by hierarchical clustering on the correlation matrix based on the cluster relative abundances as described above (Fig. 4C). A first set of samples with very similar profile composed by 3 LPS samples (7984, 11522, 8356) and one SPS sample (12924) were enriched in clusters 7, 8 and 9, which have a high expression of HLA-DR, TTF1, *beta*-catenin, and all three epithelial markers EpCAM, pan cytokeratin and cytokeratin 7. This group of ADCs is composed by the same patients that clustered together in Fig. 3C as well. Another set of ADCs composed by 3 SPS samples (13436, 13197, 12994) and one LPS sample (13376) were enriched in clusters 1, 3 and 6, which are HLA-DR and TTF1 negative. Within this group, SPS samples 13197 and 12994 were also enriched in cluster 4, which is also HLA-DR and TTF1 negative and has high vimentin expression. A last set of 2 SPS samples (13622, 12929) were enriched in clusters 5 and 10, which present high expression of vimentin, MDM2 and p-STAT3, and are negative for HLA-DR, TTF1 and *beta*-catenin. When we assessed the correlation of these epithelial clusters with the other cell types in the TME, we found that 3 clusters were significantly correlated with some immune subsets (Figs. 4D, E19). Epithelial cancer clusters 7, 8 and 9 were significantly correlated with CD4+ (r = 0.96, p < 2.2e−16; r = 0.9, p < 0.001; r = 0.78, p = 0.012) and CD8+ T cells (r = 0.95, p < 2.2e−16; r = 0.89, p = 0.0014; r = 0.76, p = 0.016). Interestingly, these specific clusters as described above, are characterized by high HLA-DR, TTF1 and *beta*-catenin, among which the former has been associated with an immunogenic profile and favorable prognosis in several cancers^21,22^.

Thus, our results show that this mass cytometry antibody panel allows the detection of subpopulations of malignant epithelial cells. Based on the cellular subsets described here, we found a high degree of intra- and inter-tumor heterogeneity. Furthermore, a significant positive correlation of HLA-DR+ ECCc with T cell infiltration and the enrichment of HLA-DR+ ECCc predominantly in LPS tumors suggests the occurrence of an immunogenic process that may be associated with a more favorable outcome.

### Validation with mIF suggests immunogenic profile in LSP tumors and RNA-Seq-based cell type enrichment analysis of independent cohort supports findings

**Figure 5:**
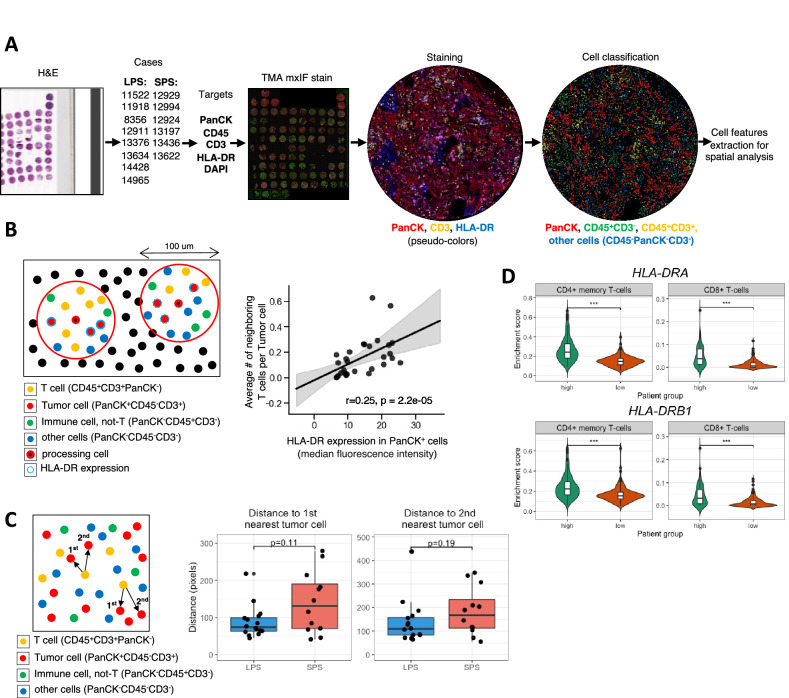
Validation by mIF on matching samples and cell enrichment analysis on RNA-Seq data from TCGA (**A**) Experiment design. TMA was generated from lung tissue blocks from patients with LPS and SPS lung adenocarcinoma. Two tissue cores were used to represent one patient. Fluorescent staining was performed for PanCK, CD45, CD3, HLA-DR, DAPI. Slides were scanned and images were extracted. Cell nuclei were segmented using deep learning algorithm (cellpose.org) and were further processed in KNIME analytical platform. Cell classification using combination of binary markers yielded following cell classes: “ECC/Tumor cells” (PanCK+CD45−CD3−), “T-cells” (CD3+CD45+PanCK−), “Immune (none-T) cells” (CD45+CD3−PanCK−), “Other cells” (CD45−CD3−PanCK−). (**B**) Correlation between HLA-DR expression on Tumor cells and T cell number was determined by Spearman’s rank-order correlation test. For this, in neighborhoods of 100 micrometers diameter for each (processing) Tumor cell, HLA-DR median fluorescence intensity in Tumor cells and average number of neighboring T cells per sample were calculated and used as inputs. (**C**) Spatial analysis was performed in KNIME by calculation of distances from each T cell to nearest 1st and 2nd Tumor cell. (**D**) Cell enrichment analysis on lung ADC RNA-Seq data from TCGA using xCell, comparing enrichment of CD4+ memory T cells and CD8+ T cells between patients with high (n = 120) vs. low (n = 120) gene expression of *HLA-DRA* and *HLA-DRB1*. Significance was assessed by Mann–Whitney U test (***p value < 0.001).

To validate our mass cytometry results and to gain insights into the spatial distribution of cellular interactions, we used mIF staining of TMA sections of lung ADC. We generated a TMA from lung tissue blocks from patients with LPS and SPS lung ADC, using two tissue cores per patient. Cases were selected to match samples analyzed by CyTOF and every tissue core was evaluated by a pathologist to ensure tissue quality (no areas of necrosis, predominant stroma or large vessels. With the exception of one patient sample (ID 7984) which stained cores were excluded due to a significant loss of material during staining, all CyTOF samples were included in this analysis along with some extra to increase statistical power. Fluorescent staining was performed for PanCK, CD45, CD3, HLA-DR, DAPI. Slides were scanned and images were extracted. Cell nuclei were segmented using deep learning algorithm (cellpose.org)^23^ and were further processed in KNIME analytical platform where cell segmentation, feature extraction and cell classification were performed^24^. Using a combination of binary markers we annotated the following cell types: “ECC/Tumor cells” (PanCK+CD45−CD3−), “T-cells” (CD3+CD45+PanCK−), “Immune (none-T) cells” (CD45+CD3−PanCK−), “Other cells” (CD45−CD3−PanCK−). Quantitative data from single cell features (such as X, Y coordinates, HLA-DR expression and etc.) was used for correlation and spatial analysis (Fig. 5A–C). We computed the correlation between HLA-DR expression on tumor cells and T cell number by Spearman’s rank-order correlation test. For this, in neighborhoods of 100 μm diameter for each (processing) tumor cell, HLA-DR median signal intensity on neighboring tumor cells and number of T cells were calculated and used as inputs for correlation analysis. We found a significant positive correlation of HLA-DR expression in tumor cells and T cell number (r = 0.25, p = 2.2e−5), confirming our previous findings (Figs. 4D, 5B). Next, spatial analysis was performed in KNIME by calculation of distances from each T cell to nearest 1st and 2nd tumor cell. T cells in LPS tumors showed a shorter distance to the first tumor cell compared to SPS tumors (Figs. 5C, E20), demonstrating that LPS tumors are more immunogenic than SPS tumors. These results support our CyTOF findings and further demonstrate by spatial analysis that LPS tumor cells are in closer proximity with T cells compared to SPS tumors, suggesting that the HLA-DR and T cell infiltration play an important role in the indolent behavior of these tumors.

Finally, acknowledging the limited sample size of our study we decided to further validate our results using the lung ADC cohort from The Cancer Genome Atlas Research Network (TCGA). In a recent study, Ma and colleagues used the same cohort and found that the top pathways associated with better prognosis were enriched for immune cell signaling-related pathways, and that MHC-II genes were among the common genes shared by these pathways^25^. When performing survival analysis they found that up-regulation of MCH-II genes was significantly associated with an improved overall survival rate. Taking these results into account, we decided to take a step further and performed cell type enrichment analysis on the same RNA-Seq data using xCell, a gene signatures-based method robustly trained and validated that identifies immune and stroma cell types^26^. When comparing samples with high vs low expression of MHC-II-related genes we found that those with high expression had significantly higher enrichment scores for multiple T cell subtypes such as CD4+ memory T cells and CD8+ T cells (Fig. 5D, Table E6). Altogether, these results provide an additional validation to our findings and highlighting the potential role of HLA-DR in tumor behavior and prognosis of lung ADC.

## Discussion

Predicting behavior of early detected ADC presents a major challenge to patients and their providers. In this study, we presented the development and validation of a mass cytometry antibody panel that aims to further our understanding of the biological determinants of early lung ADC behavior and thus improve the discrimination between indolent and aggressive tumors. First, we tested our panel in ADC cell lines and PBMC and showed that dimensionality reduction and unsupervised clustering algorithms performed optimally. We were able to accurately capture the cellular diversity between and within different cell types. Second, when we tested our panel on ten primary ADC we saw that the relative abundance of endothelial cells is positively correlated with immune cell infiltration. ADCs with LPS had a higher proportion of endothelial and immune cells, whereas a group of ADCs predicted to have SPS had higher proportion of mesenchymal cells. Third, when considering the ECC compartment, samples showed high inter- and intra-tumor heterogeneity and HLA-DR+ subpopulations were positively correlated with T cell infiltration. Specifically, a group of four samples that clustered together by cell type abundance in Fig. 3C which presented a high percentage of CD8+ and CD4+ T cells and myeloid cells, also clustered together based on their ECCc profile (Fig. 4) which was enriched in HLA-DR+ cells. Three of these samples were LPS tumors classified as stage IA or 0 cancers with small nodule size based on their CT scans (Table E4), and their histology is mostly lepidic which is associated with a favorable prognosis^27^. Conversely, the one LPS sample that deviated from this profile is a stage IB cancer, presents a bigger nodule size compared to the other LPS samples and has a predominant lepidic pattern but it also has a micropapillary component which is typically associated with a worse prognosis^27^. Finally, we validated our CyTOF findings by immunofluorescence and spatial analysis, in which we confirmed that the T cell abundance was positively correlated with HLA-DR expression in pan-cytokeratin+ cells and that T cells in LPS samples were closer to the first tumor cell in the space compared to SPS samples (Fig. 5).

The hypothesis that intra-tumor heterogeneity is associated with disease progression is not novel per se^28^. However, most studies in ADCs are based on bulk tissue analysis, which provides an average phenotype affecting the detection of rare subsets and overlooking the contribution of the TME. Single-cell technologies can overcome such limitation, providing high resolution information. Recently, the development and improvement of tissue dissociation protocols have made possible the application of single cell analysis to solid tumors^29^. A recent study using mass cytometry investigated the TME of ADC focusing on the innate immune component^13^. The authors focused on comparing blood to normal and cancer tissues, for which the latter had a higher T cell content and they identified changes in tumor infiltrating myeloid cell subpopulations that could impair anti-tumor T cell immunity. Association with clinical outcome was not reported, however. Another study used single-cell RNA Seq and obtained a deep profile of lung cancer samples, most of which were lung ADC patients, focusing on the TME and highlighting its heterogeneity and importance in tumor development^16^. Additional analysis of TCGA data showed that the abundances of some subpopulations and their correlation with patient survival differ between ADC and squamous cell carcinoma and that they were influenced by clinical characteristics such as stage. An important component of the immune response in tumor biology is played by the interaction of the major histocompatibility complex molecules class I and II. MHC-I has been widely studied in cancer and there are some pivotal publications dedicated to lung ADC specifically^30,31^. In contrast, the role of MHC-II or HLA-DR in lung ADC is less well understood. HLA-DR is constitutively expressed in antigen presenting cells but its expression can be induced in other tissues under, such as tumor cells, under inflammatory conditions^22^. Their main role is antigen presentation to CD4+ T cells, which when activated support CD8+ T cell activation and generation of memory T cells. Furthermore, tumor specific HLA-DR expression is associated with favorable outcomes in cancer patients^22^. In a recent study, Johnson and colleagues addressed the effect of HLA-DR expression in cancer cells on T cell recruitment and anti-PD1 therapy response using non-small cell lung cancer murine models^32^. They found that HLA-DR expression in cancer cells correlated with response to anti-PD1 therapy and showed by mechanistic experiments that overexpression of CIITA, a master regulator of the MHC-II pathway, in anti-PD1 resistant cells resulted in HLA-DR expression and increased T cell infiltration, whereas loss of CIITA in anti-PD1 responsive cells resulted in reduced HLA-DR expression and decreased T cell infiltration. In our data we found a strong association between HLA-DR expression in ECC and T cell abundance, mainly in LPS tumors. In addition, we found by spatial analysis an increased proximity of T cells to tumor cells in LPS tumors, suggesting that an immunogenic process could be responsible for the indolent behavior. How HLA-DR expressing ECC and closely related T cell infiltration in space contribute to the behavior of early lung ADC remains to be studied.

Our results prove mass cytometry as a suitable tool to dissect ADC biology at the single cell level and to investigate the interplay between the TME and the epithelial compartment^21,33,34^. Our work also has limitations. In this preliminary study, we are including a limited number of tumors per group (LPS, SPS) and we present these results as a proof of concept for the use of mass cytometry as a relatively novel application in ADC research. Results will be further validated in a larger cohort which is part of an ongoing study. Additionally, with this analysis we are limited to a fixed number of proteins compared to single cell RNA Seq in which thousands of transcripts can be analyzed. Yet, the latter carries the uncertainty that missing data could be non-expressed genes or non-detected genes, and for that mass cytometry data is more reliable. Additionally, protein expression of tumors presents high variability, and normal lung tissue control is not always available. We are also limited by the amount of tissue that we could collect and by the overrepresentation of SPS ADCs as we are biased towards larger lesions. As for clinical limitations, the aggressiveness and indolence of ADCs are confounded by the heterogeneous treatments patients undergo and we do not know the true natural history of early ADC. Finally, is important to consider that CANARY is not a perfect tool, and that other predictors should be consider in the future.

The difficulty in predicting behavior of early detected ADC presents a major challenge to patients and their providers. These preliminary results of mass cytometry in early lung ADC suggest a distinct cellular profile among LPS vs SPS tumors, implying an important role for T cell infiltration linked to HLA-DR expression. Future work will refine these results, integrate data from other platforms (i.e. radiomics, transcriptomics, genomics, etc.) and determine whether the combination of ECC subpopulations with specific subpopulations of cells in the TME predicts tumor behavior. We postulate that ultimately this work will allow us to better predict tumor behavior and integrate this evidence to improve current management of early lung ADCs.

## Methods

### Cell lines and cell culture

Human lung adenocarcinoma cell lines A549, PC9, H23 and Human Burkitt’s lymphoma cell line Ramos were obtained from ATCC. H3122 was provided by Dr. Christine Lovly (Vanderbilt University)^19^. Cells were grown in RPMI 1640 medium containing 10% heat-inactivated FBS (Life Technologies, cat# 16140071) and 1X Pen/Strep at 37 °C, 100% humidity, and 5% CO_2_. All cells used were in a low passage number (<5). These cell lines harbor different genetic alterations (Table E1).

### Human specimens

PBMCs were obtained from a healthy donor under an Internal Review Board (IRB) approved protocol 030763 and tumor tissues samples were collected from patients undergoing lung resection surgery following an IRB approved protocol 000616 at the Vanderbilt University Medical Center. Informed consent was obtained from all subjects. Samples were obtained from 10 lung adenocarcinoma patients, from which 5 were males and 6 were females. The ages from this patients ranged from 58 to 88 with a median of 72. See Table E2 for more details.

### Sample collection and processing

All tissue samples were processed within one hour of surgery. Lung tissues were minced, digested with Collagenase and DNase I for 1 h at 37 °C. Single-cell suspension was filtered (70 μm and 40 μm) and cryopreserved for long-term storage as previously described^29^. Cell viability was assessed before cryopreservation and after thawing. Dead cells were computationally removed as detailed in “Mass cytometry data analysis” section.

### Patient risk stratification

We analyzed the chest CT scans of the patients using a Computer-Aided Nodule Assessment and Risk Yield (CANARY) software to differentiate and stratify risk of lung adenocarcinomas^35^. CANARY analysis was performed on the CT images taken within 3 months prior surgery for all patients involved in this study. Semi-automated nodule segmentation using CANARY software detects nine classes of nodule characteristics based on voxel histogram features within the CT images which in turn helps in risk stratification of the nodule. These features are coded as Violet (V), Indigo (I), Blue (B), Green (G), Yellow (Y), Orange (O), Red (R), Cyan (C), and Pink (P). The V, I, R, O class represents solid density voxel. Classes B, C, G represent ground-glass opacity and P and Y classes indicate lepidic and invasive growth. The overall prediction of histopathological tissue invasion helps in a risk stratification of the lesions into Good (G) and Poor (P) risk groups, which we refer in the main paper as LPS and SPS, respectively. Samples were classified as shown in Table E2.

### Mass cytometry antibody panel

We have developed a comprehensive antibody panel that comprises a total of 34 antibodies, including markers for cellular lineage (immune cells, epithelial cells, endothelial cells, mesenchymal cells), cancer markers and signaling pathways. Metal-conjugated antibodies were purchased from Fluidigm and customized conjugations were performed using Maxpar Multi-Metal labeling Kits (Fluidigm) with purified antibodies from different sources (see Table 1).

### Mass cytometry sample preparation and data acquisition

Cryopreserved samples were thawed and stained with our antibody panel (Table 1) as previously described^29^. Cell lines were detached from culture flasks using TrypLE Express (Gibco) and processed following the same protocol. For intracellular staining, cells were permeabilized with methanol. To prevent cell loss, an additional fixation step was added to the protocol after the washing steps of the intracellular staining. We controlled for batch effect using EQ Four Element Calibration Beads (DVS Sciences/Fluidigm). Prior sample acquisition, cells were resuspended in 1× calibration beads in deionized water to reach a concentration of 5 × 10^5^ cells/ml. Cells were filtered using FACS tubes with filter caps (Corning Falcon) and collected using a standard/narrow bore on a Helios CyTOF system at the Mass Cytometry Center of Excellence at Vanderbilt University. The number of events acquired is specified in Table E3.

#### Cell lines

To validate our antibody panel we used four ADC cell lines (Table E1) and PBMCs from a healthy donor. In one experiment, we pooled and stained the 4 cell lines and PBMCs in the same proportions (0.5 million cells per group) and we repeat this experiment. In other experiment, we stained and run the different cell groups separately (1 million cells per group). All cells were stained with the same panel (Table 1) and we used Histone H3 expression to identify nucleated intact cells.

#### Human samples

Patient samples were stained and processed in the same fashion as cell lines. Batches are described in Table E3. For every batch, a control was stained and run on the same day. This control was a mixture of A549 and Ramos cells, 1 million cells of each.

### Mass cytometry data analysis

#### Data preprocessing

Collected events from both validation experiments with cell lines and human samples were processed in the same fashion. Prior to analysis, all mass cytometry FCS files were normalized using the premessa R package (https://github.com/ParkerICI/premessa, version 0.2.4), an R implementation of the MATLAB bead normalization software^36^. Normalized data was initially analyzed in Cytobank^37^. Noise reduction parameters were as follows: cells with Histone H3 < 10 were considered dead and excluded, only cells with an event length 10–70 were considered singlets and included.

#### Cell lines

For data shown in Figs. 1, 2 we used the data acquired for each cell line individually, performed random equal subsampling (15,000 events per sample), and concatenated the files. UMAP plots shown in Figs. 1, 2 were generated in R using all markers of Table 1, except for Histone H3. We used k-means for clustering analysis and applied the same markers. To determine the optimal number of clusters *k* to target, we used the ’elbow’ criterion, for which the total within-cluster sum of squares was calculated for a range of values of *k*^20^. Clustering was performed with *k* = 8.

#### Human samples

To determine cellular identity, we performed k-means using markers that identify main cellular populations (EpCAM, CD31, CD45, vimentin, cytokeratin and cytokeratin7). We targeted for a large number of clusters (*k* = 10) to allow for more granularity and prevent rare cell populations from being engulfed into dominant clusters. These were annotated based on protein expression and clusters with similar characteristics were merged. Final cell types were annotated as epithelial cancer cells, endothelial cells, mesenchymal cells and immune cells. Epithelial cancer cells were defined as EpCAM+/cytokeratin+/cytokeratin7+, endothelial cells as CD45−/CD31+, mesenchymal cells as vimentin+/CD45−/CD31−/EpCAM−/cytokeratin−/cytokeratin7− and immune cells as CD45+. We performed a second clustering round for immune cells only (*k* = 10) using immune cell markers CD8, CD24, CD3, CD11b, CD56 and HLA-DR. Cluster were annotated into myeloid cells (CD45+/CD3−/CD11b+), cytotoxic T cells (CD45+/CD3+/CD8+), helper T cells (CD45+/CD3+/CD4+) and other immune as the remaining CD45+ cells. Figure 3A is a representation of the annotated cell types of the 10 tumors using the same markers from the two clustering rounds to generate the UMAP plots, for which we obtained a random sample without replacement for a total of 4000 events per sample. Epithelial cancer cells from each entire sample were subseted and clustered using k-means (*k* = 10) and the following markers: EpCAM, c-casp3, TP53, HLA-DR, HLA-ABC, CD31, thioredoxin, *beta*-catenin, HER2, p-STAT3, p-STAT5, p-STAT6, TTF1, p-AKT, Ki67, CD56, vimentin, MDM2, cytokeratin, MET, TP63, CK7, EGFR, CD44, p-ERK, CD24, p-S6, PDL1. Figure 4A is a representation of the clusters of the 10 tumors using the same markers from the previous clustering to generate the UMAP plots, with random sampling without replacement for for a total of 2000 events per sample.

### Multiplex immunofluorescence validation of CyTOF data

#### Tissue microarray

TMA was generated from lung tissue blocks from patients with LPS and SPS lung adenocarcinoma. Two tissue cores were used to represent one patient. First, specific cases were selected to match samples, analyzed by CytOF, next, every core was evaluated by pathologist to ensure tissue quality (no massive areas with necrosis, stroma, large vessels; no processing artefacts).

#### Staining

TMA paraffin blocks were cut into 5 μm sections. Hematoxylin Eosin staining was used for visual evaluation of morphology to ensure comparable tissue samples were used for analysis. Multiplexed Immunofluorescent (mxIF) stain was performed with following antibodies: anti-PanCK, Clone AE1/AE3 (Invitrogen); anti-CD45, Clone HI30 (Biolegend); anti-CD3 (Agilent Inc., Dako); anti-HLADR, Clone SPM288 (Novus Biologicals LLC.). Multistep mxIF staining was perform, where after blocking, in a first step tissue was incubated with mouse anti-CD45 antibodies, followed by Fab fragment anti-mouse-Cy3 (Jackson ImmunoResearch). Tissue was washed well to remove unbound antibodies, blocked with mouse IgG and incubated with directly conjugated mouse PanCK-FITC, HLADR-Cy7 and rabbit anti-CD3 antibodies. Next, after washing, CD3 was detected in additional step with anti-rabbit-Cy5 (Thermo Fisher Scientific) antibodies. Nuclei were stained with DAPI (Thermo Fisher Scientific). Slides were coverslip with prolong gold (Invitrogen) and dried overnight. Whole slide imaging was performed on Aperio Versa 200 (Leica) scanner.

#### Single cell analysis

To perform single cell analysis of multiplexed fluorescent stained images, image analysis pipeline was built in KNIME (Knime.com) analytical platform (KNIME 4.1.2 with integrated image processing and analysis extensions)^24,38^. DAPI-stained images were used to generate nuclear masks using deep learning algorithm^23^. Cell segmentation was generated by circular outgrow of nuclear masks. Single cell features were extracted by aligning nuclear or cell masks to specific fluorescent stain images. Geometrical, statistical, and texture features were extracted for each segmented cell. For cell classifications, training set of positive and negative cells was annotated. These annotations along with extracted from each specific stain features, were used for machine learning where XG boost AI models were generated for each marker. These models were applied to whole data set and resulting probabilities with p $\ge$ 0.9 cutoff were used for initial binary cell classification: “PanCK+ or PanCK−” “CD45+ or CD45−” “CD3+ or CD3−”. Cell classification using combination of binary markers yielded following cell classes: “Epithelial/Tumor cells” (PanCK+CD45−CD3−), “T-cells” (CD3+CD45+PanCK−), “Immune (none-T) cells” (CD45+CD3−PanCK−), “Other cells” (CD45−CD3−PanCK−). Quantitative data from single cell features (such as X, Y coordinates, HLA-DR expression and etc.) was used for correlation and spatial analysis. Continuous scale of fluorescent signal was used to quantify HLA-DR expression on tumor cells. For this, signal intensities normalized to DAPI (sums fluorescent signals) were used. Total cell number and specific class cell number per image were quantified and percent calculations were made. Correlation between HLA-DR expression on Tumor cells and T cell number was determined by Spearman’s rank-order correlation test. In neighborhoods of 100 μm diameter for each (processing) Tumor cell, HLA-DR median signal intensity on neighboring Tumor cells and number of T cells were calculated in Python and used as inputs for correlation analysis. Spatial analysis was performed in KNIME by calculation of distances from each T cell to nearest 1st and 2nd Tumor cell using similarity search node.

### TCGA lung ADC data set

Fragments Per Kilobase of transcript per Million (FPKM) normalized read counts of RNA-Seq from lung ADC patients and matching clinical data were downloaded from National Cancer Institute Genomic Data Commons Data Portal (https://portal.gdc.cancer.gov/projects/TCGA-LUAD).

### Cell type enrichment analysis with xCell

Using TCGA data, we selected patients with disease stage between I and III. After applying log transformation ($log_{2}(FPKM + 1)$) we computed the quantiles of expression of MHC-II related genes. Patients were labeled as “low” if the expression of the gene in question was below the first quantile (25%) and “high” if it was higher than the third quantile (75%). Cell type enrichment analysis results for TCGA data were downloaded from the xCell website (https://xcell.ucsf.edu/) and patient groups were compared.

### Statistical analysis

For correlation analysis we used Spearman’s rank correlation test and adjusted p-values for multiple hypothesis using the Benjamini and Hochberg method^39^. Comparison of categorical variables was performed using the Mann–Whitney U test. Survival curves were generated using the Kaplan–Meier method, and statistically significant differences were analyzed with the log rank test. All statistical tests were two-sided and p values less than 0.05 were considered statistically significant.

## Supplementary Information

Supplementary Information.

## Data Availability

Further information and requests for resources and data should be directed to and will be provided by the corresponding author. All methods were carried out in accordance with relevant guidelines and regulations.
