# Supplementary material for: HLA-DR cancer cells expression correlates with T cell infiltration and is enriched in lung adenocarcinoma with indolent behavior
Source: Sci Rep. 2021 Jul 13;11:14424. doi: 10.1038/s41598-021-93807-3 (PMC8277797; doi:10.1038/s41598-021-93807-3)
Supplement: Supplementary file 1 — Supplementary Information. [file 41598_2021_93807_MOESM1_ESM.pdf]

Supplementary Information

HLA-DR cancer cells expression correlates with T cell infiltration and is enriched in lung adenocarcinoma with indolent behavior

Maria-Fernanda Senosain[1,2,3,*], Yong Zou[2,3], Tatiana Novitskaya[7], Georgii Vasiukov[2,7], Aneri B. Balar[2,3], Dianna J. Rowe[2,3], Deon B. Doxie[4,5], Jonathan M. Lehman[6], Rosana Eisenberg[7], Fabien Maldonado[2], Andries Zijlstra[7], Sergey V. Novitskiy[7], Jonathan M. Irish[4,5,7], and Pierre P. Massion[2,3,8,†]

[1]Cancer Biology Graduate Program, Vanderbilt University, Nashville, TN, USA
[2]Division of Allergy, Pulmonary, and Critical Care Medicine, Department of Medicine, Vanderbilt University Medical Center, Nashville, TN, USA
[3]Cancer Early Detection and Prevention Initiative, Vanderbilt-Ingram Cancer Center, Vanderbilt University Medical Center, Nashville, TN, USA
[4]Vanderbilt-Ingram Cancer Center, Vanderbilt University Medical Center, Nashville, TN, USA
[5]Department of Cell and Developmental Biology, Vanderbilt University, Nashville, TN, USA
[6]Division of Hematology/Oncology, Department of Medicine, Vanderbilt University Medical Center, Nashville, TN, USA
[7]Department of Pathology, Microbiology and Immunology, Vanderbilt University Medical Center, Nashville, TN, USA
[8]US Department of Veterans Affairs, Tennessee Valley Healthcare System, Nashville, TN, USA
[†]Deceased
[*]mariafernanda.senosain@vanderbilt.edu

Cell line	Genetic Alteration
A549	
	KRAS activating mutation
	CDKN2A locus deletion
H3122	
	EML4-ALK variant 1, activating mutation
PC9	
	EGFR activating mutation
	TP53 inactivating mutation
H23	
	TP53 inactivating mutation
	KRAS activating mutation

Table E1. Lung ADC Cell lines genomic profiles

Characteristic		Patients (N=10)
Sex		
	Male	4
	Female	6
Age		
	Median	68.5
	Range	56 - 86
Race		
	Caucasian	10
Smoking status		
	Smoker	1
	Ex-smoker	9
Family history of cancer		
	Lung	2
	Other	5
Nodule size (mm)		
	Median	31.5
	Range	9.7 - 61
Pathological Stage		
	Stage 0	1
	Stage IA	2
	Stage IB	1
	Stage IIA	1
	Stage IIB	4
	Stage IIIB	1
Tumor location		
	RLL	3
	RUL	4
	LLL	3
Risk stratification (CANARY)		
	LPS	4
	SPS	6

Table E2. Summarized patient characteristics (CyTOF only)

Patient ID	CANARY	Batch ID	Batch #	Events*
7984	LPS	32618	4	16787
8356	LPS	32118	2	7471
11522	LPS	32418	3	8194
12924	SPS	32618	4	255991
12929	SPS	32418	3	48359
12994	SPS	32118	2	104147
13197	SPS	32418	3	51198
13376	LPS	31618	1	653176
13436	SPS	31618	1	32681
13622	SPS	32618	4	501184

Table E3. CyTOF Sample batches. **Number of events (cells) after pre-processing.*

Pt ID	CANARY	Age at collection	Sex	Race	Smoking Status	Age Started	Age Quit	Pack Years	Family History Cancer Type	CT Nodule Size (mm)	CT Nodule Location	8th Edition Path Stage	Biological data
7984	LPS	66	Female	Caucasian	Ex-smoker	15	69	50	Unknown	9.7	RLL	Stage IA1	CyTOF
8356	LPS	72	Female	Caucasian	Ex-smoker	18	60	37	Pancreatic	23.4	LLL	Stage 0	Both
11522	LPS	57	Female	Caucasian	Ex-smoker	16	57	20.5	Unknown	28	RUL	Stage IA3	Both
12924	SPS	70	Male	Caucasian	Ex-smoker	17	70	53	Melanoma Skin Cancer	31	LLL	Stage IIB	Both
12929	SPS	86	Female	Caucasian	Ex-smoker	16	41	37.5	Unknown	37	LLL	Stage IIB	Both
12994	SPS	76	Male	Caucasian	Ex-smoker	20	44	24	Brain	60	RUL	Stage IIIB	Both
13197	SPS	78	Male	Caucasian	Ex-smoker	20	55	35	Lung Cancer	13	RLL	Stage IIB	Both
13376	LPS	64	Female	Caucasian	Current smoker	16	N/A	20	Bladder	41	RUL	Stage IB	Both
13436	SPS	56	Male	Caucasian	Ex-smoker	21	51	45	Gynecological Cancer	61	RLL	Stage IIB	Both
13622	SPS	67	Female	Caucasian	Ex-smoker	12	67	110	Lung Cancer	32	RUL	Stage IIA	Both
11918	LPS	68	Male	African American	Ex-smoker	18	43	25	Gastrointestinal Cancer	22	RUL	Stage IA1	MxIF
12911	LPS	72	Male	African American	Ex-smoker	31	61	15	Unknown	12	LUL	Stage IA2	MxIF
13634	LPS	67	Female	Caucasian	Current Smoker	13	N/A	N/A	Other	N/A	RUL	Stage IIIB	MxIF
14428	LPS	73	Male	Caucasian	Current Smoker	N/A	N/A	45	Gynecological Cancer	38	RUL	Stage IA2	MxIF
14965	LPS	62	Female	Caucasian	Never smoker	N/A	N/A	N/A	Other	N/A	LUL	Stage IA3	MxIF

Table E4. Detailed patient clinical characteristics

Pt ID	CANARY	Solid	Acinar	Lepidic	Mucinous	Micropapillary
7984	LPS		+++	+		
8356	LPS			+++		
11522	LPS			+++		
12924	SPS	+	+	+		
12929	SPS	+	+++			
12994	SPS		+++			+
13197	SPS		+++			+
13376	LPS		+	+++		+
13436	SPS		+++	+	+++	+
13622	SPS	+				

Table E5. Histologic subtypes of ADC

Gene	Cell type	Size.high	Size.low	Median.High	Median.Low	p.value	p.adjusted
HLA.DRA	CD4+ memory T-cells	120	120	0.2415	0.1475	1.05E-18	3.93E-18
HLA.DRA	CD4+ naive T-cells	120	120	0.10225	0.02488	6.61E-24	4.96E-23
HLA.DRA	CD8+ naive T-cells	120	120	0.0067875	0.005175	0.16879963	0.19781206
HLA.DRA	CD8+ T-cells	120	120	0.037765	0.006478	2.23E-13	5.08E-13
HLA.DRA	CD8+ Tcm	120	120	0.05113	0.008943	6.63E-26	8.29E-25
HLA.DRB5	CD4+ memory T-cells	120	120	0.2002	0.15705	5.77E-05	7.46E-05
HLA.DRB5	CD4+ naive T-cells	120	120	0.08357	0.028725	5.51E-17	1.53E-16
HLA.DRB5	CD8+ naive T-cells	120	120	0.0053235	0.005488	0.93774746	0.96609699
HLA.DRB5	CD8+ T-cells	120	120	0.022435	0.0100045	7.07E-05	8.99E-05
HLA.DRB5	CD8+ Tcm	120	120	0.034265	0.010875	6.01E-12	1.13E-11
HLA.DRB6	CD4+ memory T-cells	120	120	0.21425	0.16055	6.07E-08	9.10E-08
HLA.DRB6	CD4+ naive T-cells	120	120	0.08662	0.026945	1.04E-12	2.10E-12
HLA.DRB6	CD8+ naive T-cells	120	120	0.006645	0.005613	0.65471749	0.70148302
HLA.DRB6	CD8+ T-cells	120	120	0.03306	0.011785	7.97E-07	1.13E-06
HLA.DRB6	CD8+ Tcm	120	120	0.04045	0.01496	4.07E-13	8.98E-13
HLA.DRB1	CD4+ memory T-cells	120	120	0.2232	0.1545	1.01E-09	1.69E-09
HLA.DRB1	CD4+ naive T-cells	120	120	0.100035	0.02494	6.49E-23	4.06E-22
HLA.DRB1	CD8+ naive T-cells	120	120	0.005806	0.005212	0.23544515	0.27166748
HLA.DRB1	CD8+ T-cells	120	120	0.032355	0.006944	3.71E-09	5.80E-09
HLA.DRB1	CD8+ Tcm	120	120	0.04474	0.0102135	1.12E-17	3.49E-17
HLA.DQA1	CD4+ memory T-cells	119	120	0.2328	0.1486	7.12E-13	1.48E-12
HLA.DQA1	CD4+ naive T-cells	119	120	0.09937	0.025295	2.78E-24	2.60E-23
HLA.DQA1	CD8+ naive T-cells	119	120	0.005452	0.005777	0.97313001	0.97313001
HLA.DQA1	CD8+ T-cells	119	120	0.02962	0.0100045	1.17E-07	1.73E-07
HLA.DQA1	CD8+ Tcm	119	120	0.04678	0.0092275	1.13E-20	5.28E-20
HLA.DQB1	CD4+ memory T-cells	118	120	0.2117	0.1659	2.71E-05	3.57E-05
HLA.DQB1	CD4+ naive T-cells	118	120	0.09492	0.02308	5.31E-22	3.06E-21
HLA.DQB1	CD8+ naive T-cells	118	120	0.005436	0.0053665	0.91751113	0.96609699
HLA.DQB1	CD8+ T-cells	118	120	0.02703	0.00977	3.78E-06	5.16E-06
HLA.DQB1	CD8+ Tcm	118	120	0.03978	0.01308	7.20E-14	1.74E-13
HLA.DQA2	CD4+ memory T-cells	120	120	0.2308	0.149	1.15E-13	2.70E-13
HLA.DQA2	CD4+ naive T-cells	120	120	0.08491	0.02863	2.99E-17	8.98E-17
HLA.DQA2	CD8+ naive T-cells	120	120	0.0055865	0.0053505	0.96662672	0.97313001
HLA.DQA2	CD8+ T-cells	120	120	0.031205	0.009417	3.61E-09	5.76E-09
HLA.DQA2	CD8+ Tcm	120	120	0.047205	0.014475	8.83E-18	2.88E-17
HLA.DQB2	CD4+ memory T-cells	117	120	0.1855	0.15905	0.00865005	0.01081256
HLA.DQB2	CD4+ naive T-cells	117	120	0.09023	0.02578	1.32E-18	4.70E-18
HLA.DQB2	CD8+ naive T-cells	117	120	0.005838	0.004884	0.25157037	0.28160863
HLA.DQB2	CD8+ T-cells	117	120	0.02652	0.0076725	5.58E-06	7.48E-06
HLA.DQB2	CD8+ Tcm	117	120	0.03031	0.011095	1.41E-09	2.31E-09
HLA.DOB	CD4+ memory T-cells	120	120	0.2475	0.1467	8.62E-18	2.88E-17
HLA.DOB	CD4+ naive T-cells	120	120	0.1095	0.02623	3.79E-25	4.06E-24
HLA.DOB	CD8+ naive T-cells	120	120	0.006561	0.005043	0.11101426	0.13215983
HLA.DOB	CD8+ T-cells	120	120	0.041405	0.0056895	2.12E-19	8.39E-19
HLA.DOB	CD8+ Tcm	120	120	0.056605	0.0078265	3.51E-27	6.57E-26
HLA.DMB	CD4+ memory T-cells	119	120	0.2486	0.1475	7.56E-20	3.15E-19
HLA.DMB	CD4+ naive T-cells	119	120	0.1043	0.024645	1.82E-21	9.73E-21
HLA.DMB	CD8+ naive T-cells	119	120	0.006214	0.0054535	0.52646757	0.58066276
HLA.DMB	CD8+ T-cells	119	120	0.04049	0.007085	6.30E-13	1.35E-12
HLA.DMB	CD8+ Tcm	119	120	0.05465	0.008451	1.66E-26	2.48E-25
HLA.DMA	CD4+ memory T-cells	120	120	0.2063	0.15705	2.67E-06	3.71E-06
HLA.DMA	CD4+ naive T-cells	120	120	0.090215	0.02454	3.55E-23	2.42E-22
HLA.DMA	CD8+ naive T-cells	120	120	0.0062675	0.0054115	0.24704118	0.28072861
HLA.DMA	CD8+ T-cells	120	120	0.029275	0.0072795	3.88E-09	5.94E-09
HLA.DMA	CD8+ Tcm	120	120	0.033825	0.01038	5.33E-17	1.53E-16
HLA.DOA	CD4+ memory T-cells	120	119	0.2183	0.1504	2.64E-11	4.61E-11
HLA.DOA	CD4+ naive T-cells	120	119	0.10365	0.02488	1.27E-28	4.75E-27
HLA.DOA	CD8+ naive T-cells	120	119	0.005298	0.006384	0.05667027	0.06855275
HLA.DOA	CD8+ T-cells	120	119	0.0355	0.009776	3.64E-10	6.21E-10
HLA.DOA	CD8+ Tcm	120	119	0.041985	0.009255	2.91E-20	1.28E-19
HLA.DPA1	CD4+ memory T-cells	119	120	0.233	0.14855	4.20E-15	1.05E-14
HLA.DPA1	CD4+ naive T-cells	119	120	0.1043	0.022955	1.48E-27	3.70E-26
HLA.DPA1	CD8+ naive T-cells	119	120	0.005774	0.0053435	0.9403344	0.96609699
HLA.DPA1	CD8+ T-cells	119	120	0.03645	0.007038	1.13E-11	2.07E-11
HLA.DPA1	CD8+ Tcm	119	120	0.05308	0.007936	4.79E-24	3.99E-23
HLA.DPB1	CD4+ memory T-cells	120	120	0.23745	0.15055	2.00E-12	3.94E-12
HLA.DPB1	CD4+ naive T-cells	120	120	0.1132	0.024285	1.18E-29	8.82E-28
HLA.DPB1	CD8+ naive T-cells	120	120	0.006382	0.006102	0.64400735	0.70000798
HLA.DPB1	CD8+ T-cells	120	120	0.040475	0.0073185	3.08E-12	5.92E-12
HLA.DPB1	CD8+ Tcm	120	120	0.052265	0.0092275	6.63E-21	3.31E-20
HLA.DPB2	CD4+ memory T-cells	120	119	0.23275	0.1523	1.74E-11	3.11E-11
HLA.DPB2	CD4+ naive T-cells	120	119	0.089785	0.03013	6.64E-16	1.72E-15
HLA.DPB2	CD8+ naive T-cells	120	119	0.007628	0.005168	0.0457618	0.05626451
HLA.DPB2	CD8+ T-cells	120	119	0.03291	0.009776	3.98E-07	5.74E-07
HLA.DPB2	CD8+ Tcm	120	119	0.048415	0.01312	5.91E-16	1.58E-15

Table E6. Summary of cell type enrichment analysis on ADC TCGA using xCell.

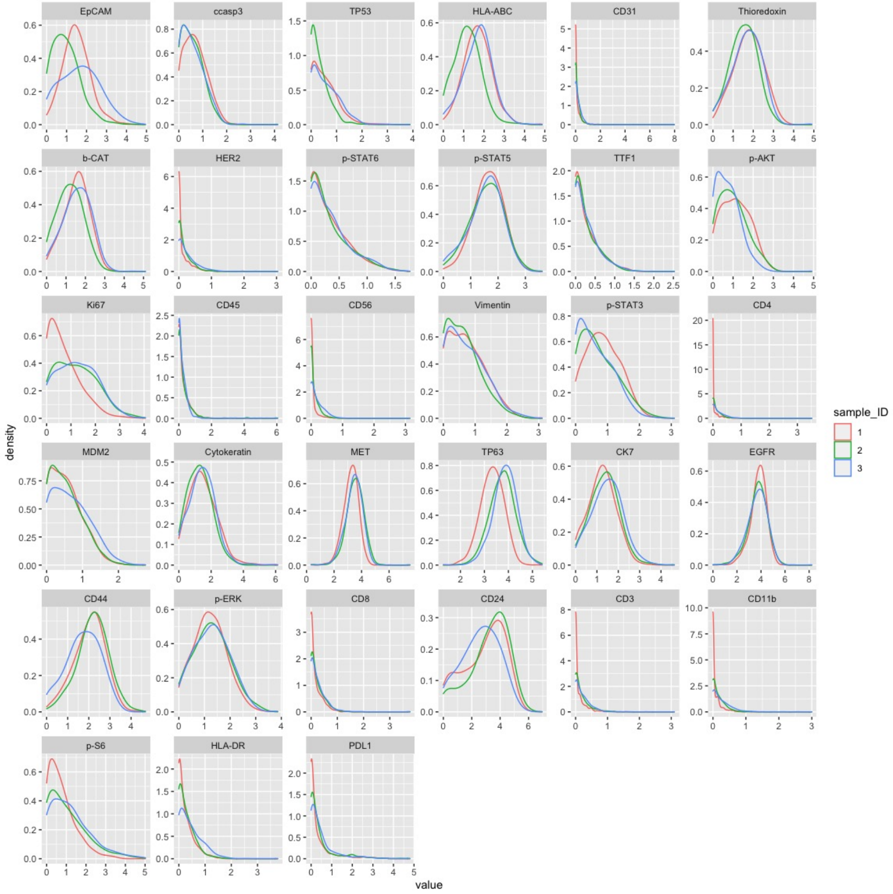

Figure E1. A549 protein expression across replicates. Samples 1 and 2 correspond to experimental mix of cell lines stained and run through the CyTOF machine separately. Sample 3 corresponds to a computational mix, for which each cell line and PBMCs were stained and run through the CyTOF machine independently and then files were concatenated to obtain a labeled mix. All samples were analyzed in Cytobank, where cell types were manually gated and annotated based on protein expression. This is the data for A549 cell line.

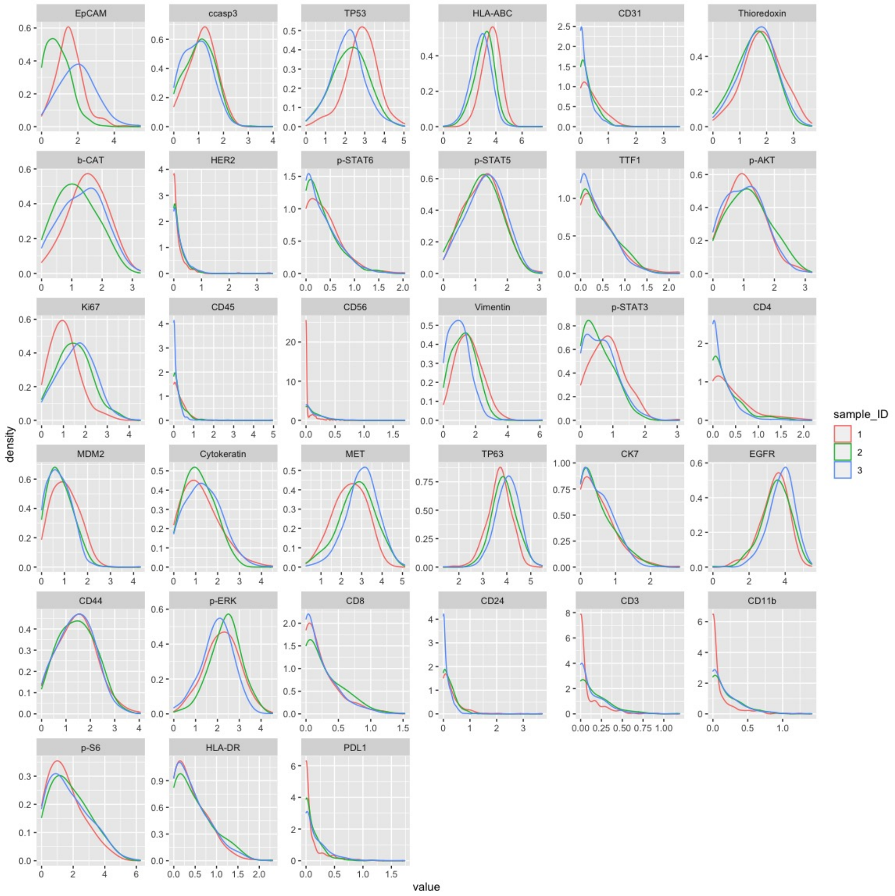

Figure E2. H23 protein expression across replicates. Samples 1 and 2 correspond to experimental mix of cell lines stained and run through the CyTOF machine separately. Sample 3 corresponds to a computational mix, for which each cell line and PBMCs were stained and run through the CyTOF machine independently and then files were concatenated to obtain a labeled mix. All samples were analyzed in Cytobank, where cell types were manually gated and annotated based on protein expression. This is the data for H23 cell line.

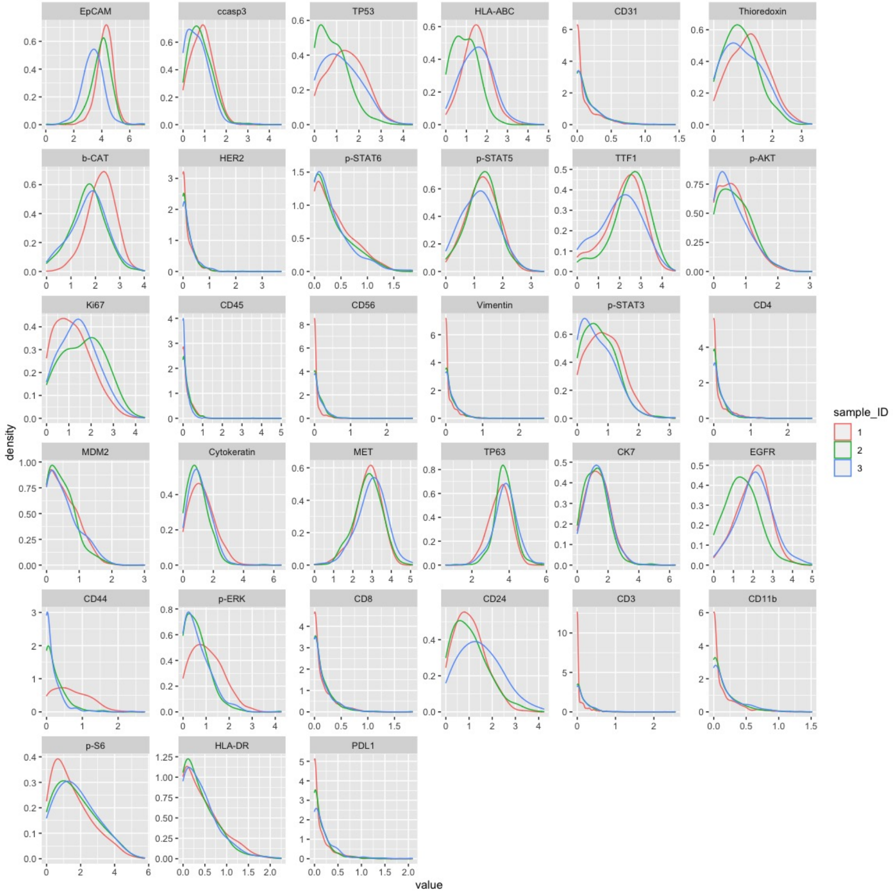

Figure E3. H3122 protein expression across replicates. Samples 1 and 2 correspond to experimental mix of cell lines stained and run through the CyTOF machine separately. Sample 3 corresponds to a computational mix, for which each cell line and PBMCs were stained and run through the CyTOF machine independently and then files were concatenated to obtain a labeled mix. All samples were analyzed in Cytobank, where cell types were manually gated and annotated based on protein expression. This is the data for H3122 cell line.

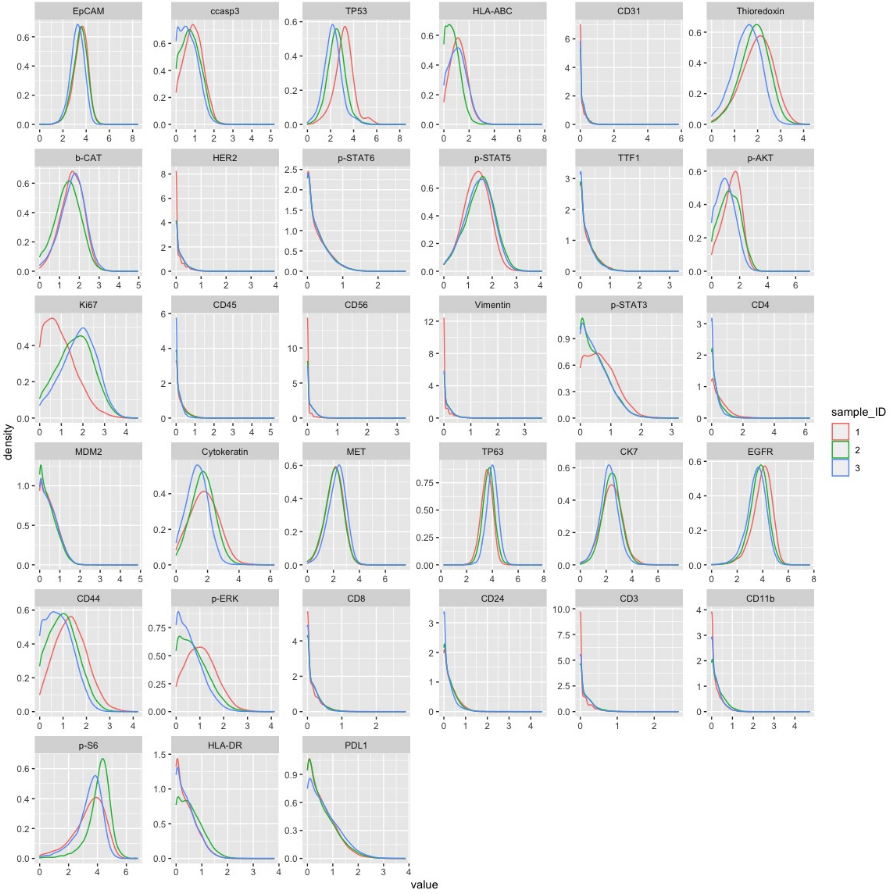

Figure E4. PC9 protein expression across replicates. Samples 1 and 2 correspond to experimental mix of cell lines stained and run through the CyTOF machine separately. Sample 3 corresponds to a computational mix, for which each cell line and PBMCs were stained and run through the CyTOF machine independently and then files were concatenated to obtain a labeled mix. All samples were analyzed in Cytobank, where cell types were manually gated and annotated based on protein expression. This is the data for PC9 cell line.

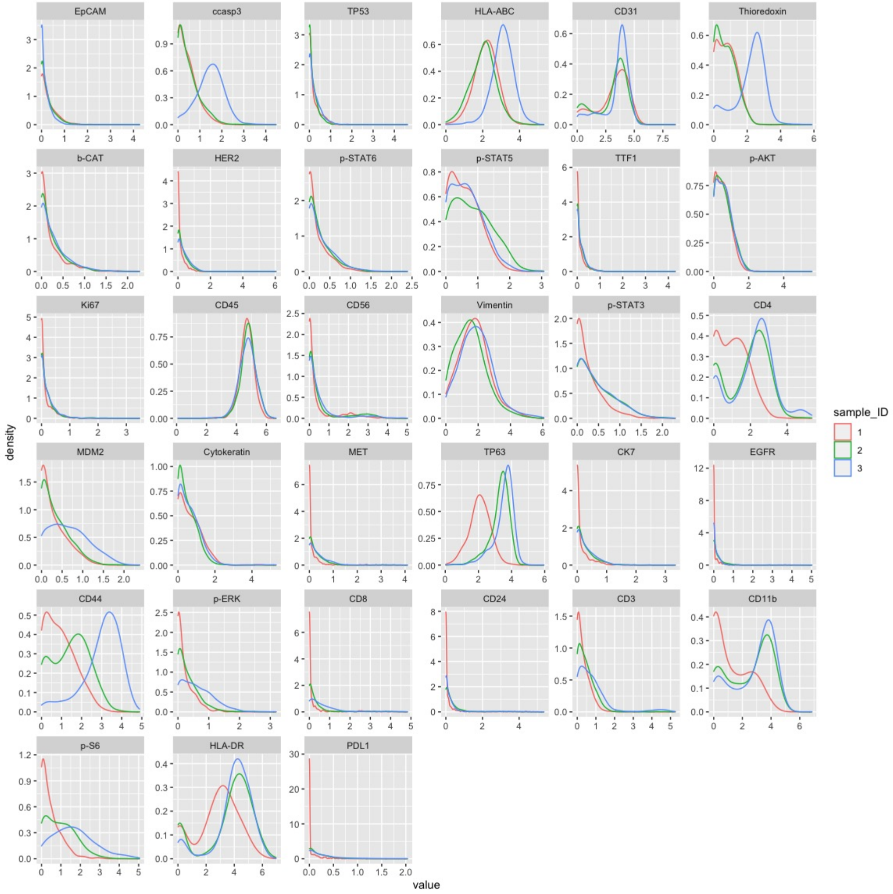

Figure E5. Monocytes protein expression across replicates. Samples 1 and 2 correspond to experimental mix of cell lines stained and run through the CyTOF machine separately. Sample 3 corresponds to a computational mix, for which each cell line and PBMCs were stained and run through the CyTOF machine independently and then files were concatenated to obtain a labeled mix. All samples were analyzed in Cytobank, where cell types were manually gated and annotated based on protein expression. This is the data for monocytes from the PBMC sample.

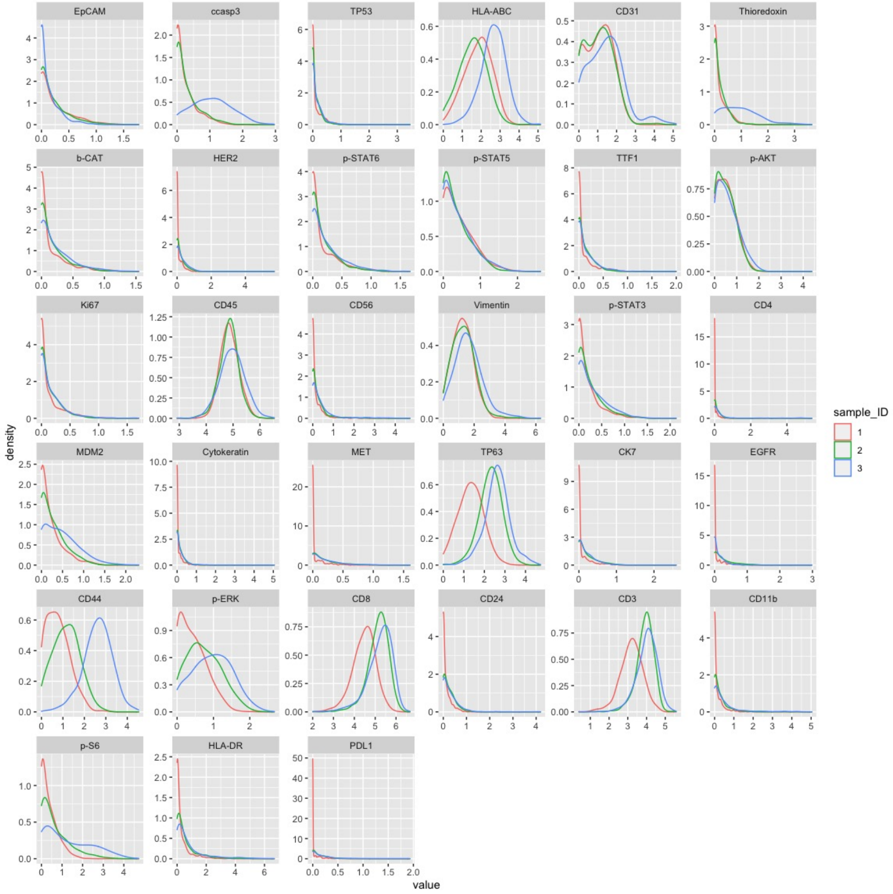

Figure E6. Cytotoxic T cells protein expression across replicates. Samples 1 and 2 correspond to experimental mix of cell lines stained and run through the CyTOF machine separately. Sample 3 corresponds to a computational mix, for which each cell line and PBMCs were stained and run through the CyTOF machine independently and then files were concatenated to obtain a labeled mix. All samples were analyzed in Cytobank, where cell types were manually gated and annotated based on protein expression. This is the data for CD8+ T cells from the PBMC sample.

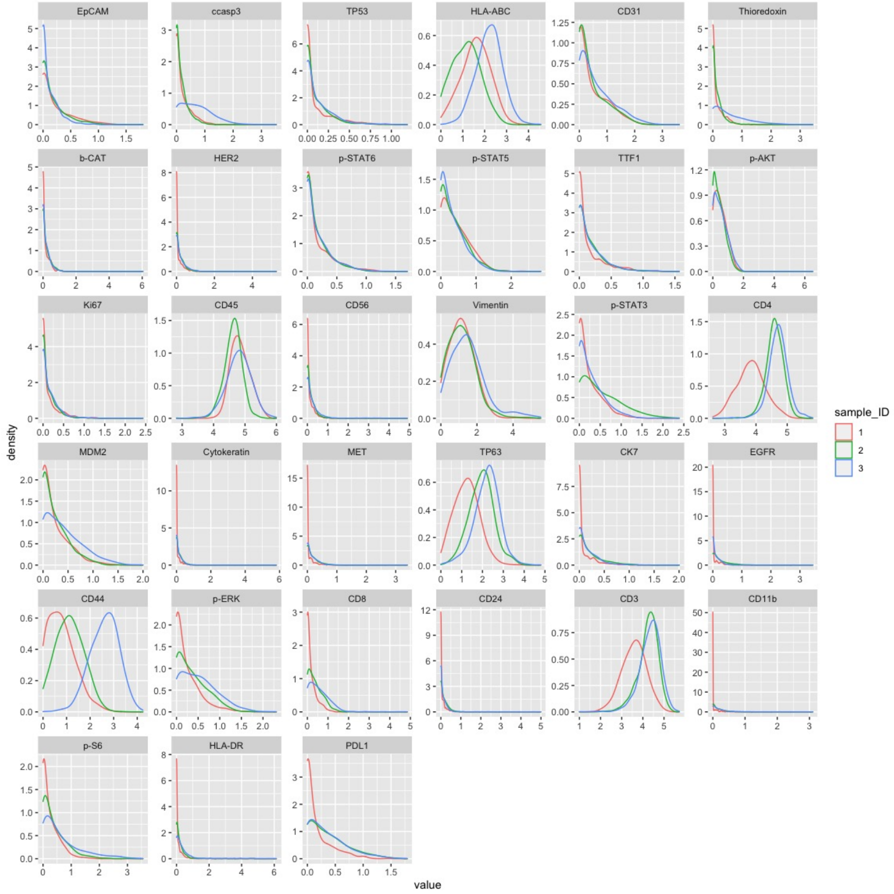

Figure E7. T helper cells protein expression across replicates. Samples 1 and 2 correspond to experimental mix of cell lines stained and run through the CyTOF machine separately. Sample 3 corresponds to a computational mix, for which each cell line and PBMCs were stained and run through the CyTOF machine independently and then files were concatenated to obtain a labeled mix. All samples were analyzed in Cytobank, where cell types were manually gated and annotated based on protein expression. This is the data for CD4+ T cells from the PBMC sample.

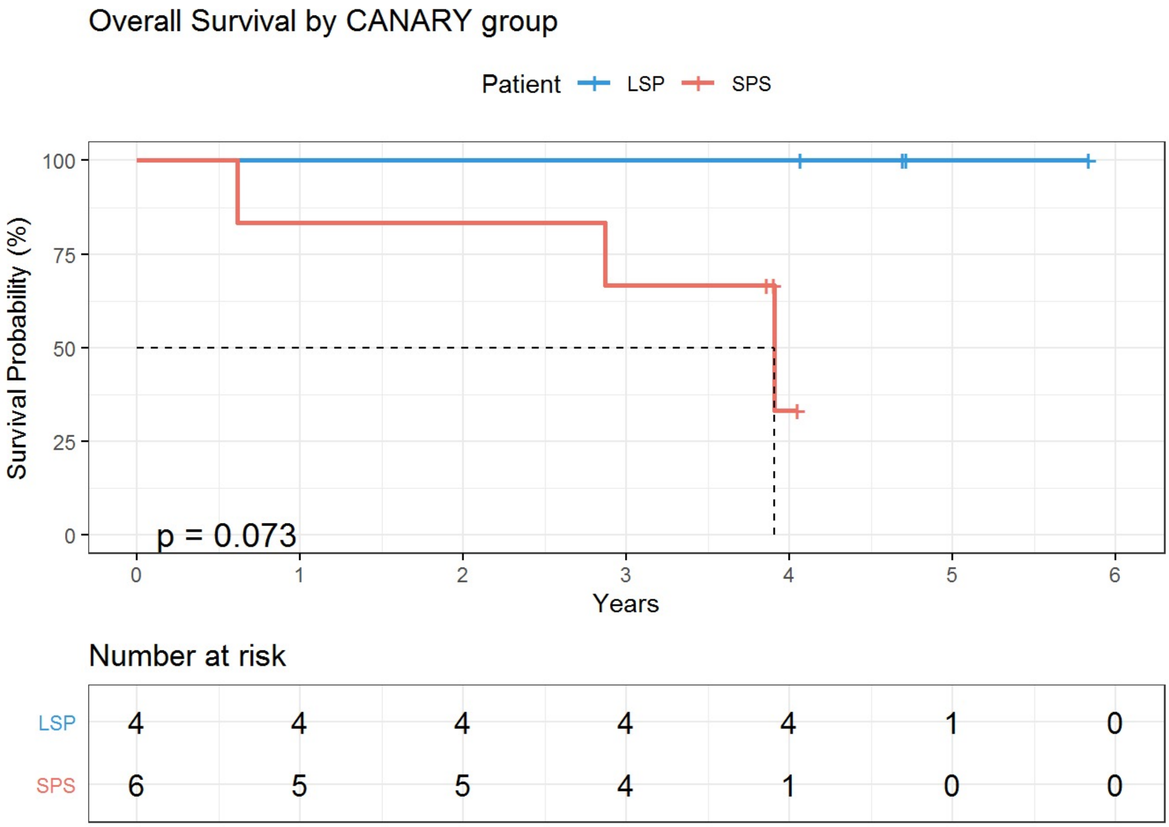

Figure E8. Survival analysis of LPS vs SPS ADC samples. Survival curves were generated using the Kaplan-Meier method, and statistically significant differences were analyzed with the log rank test.

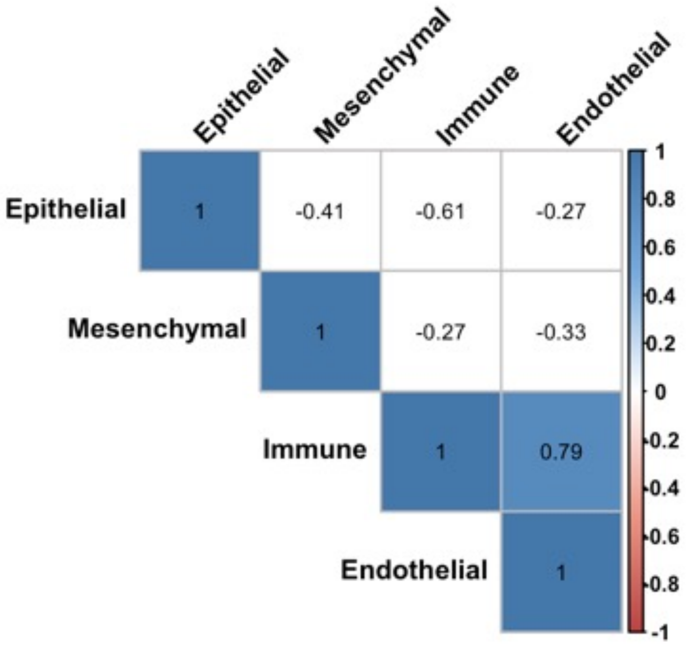

Figure E9. Spearman correlation of main cell types. Only significant correlations (p value >0.05) are colored. P values are adjusted for multiple hypothesis testing by Benjamini-Hochberg procedure.

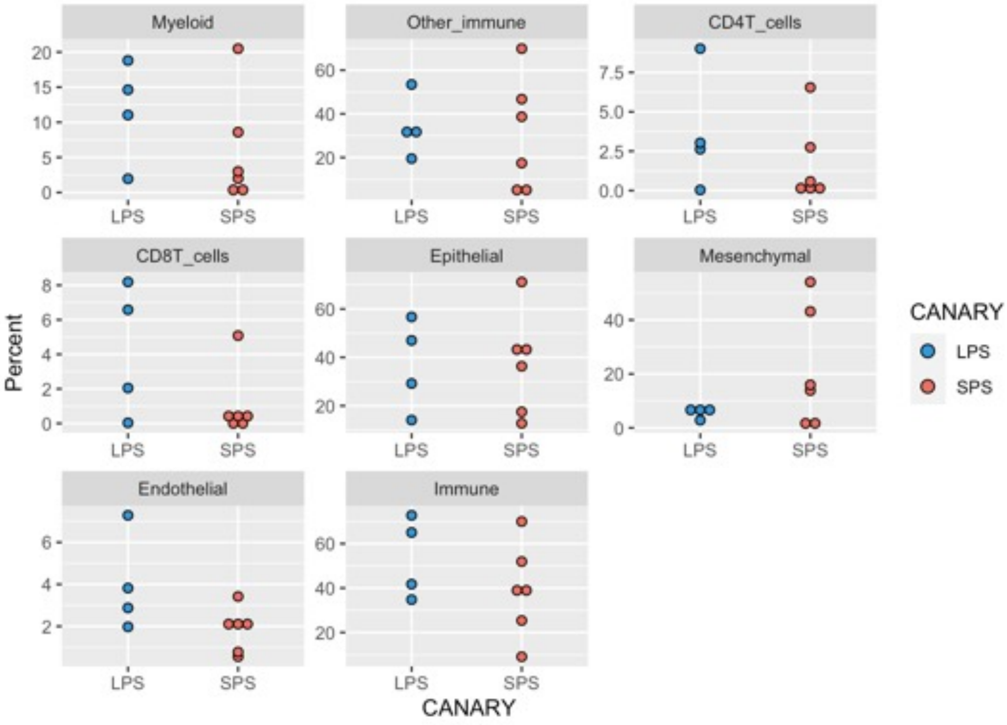

Figure E10. Differential abundance analysis. P value >0.05 for all comparisons. "Immune" correspond to the percentages of all immune subtypes added up per patient.

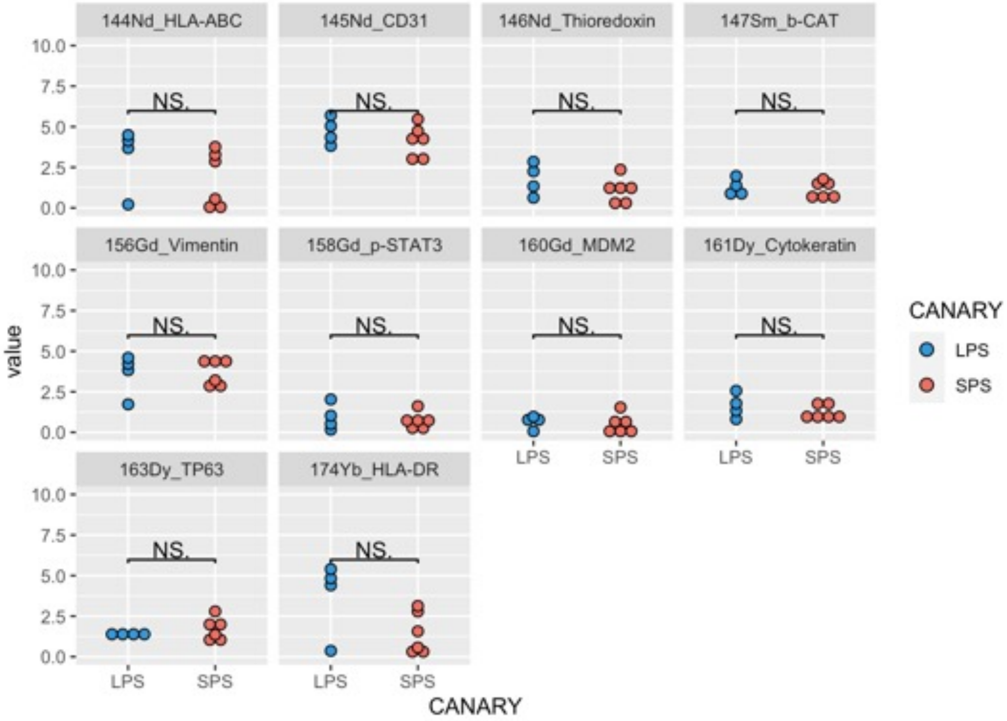

Figure E11. Protein expression comparison for endothelial cells. Only the protein markers which have an average protein expression > 1.4 for at least one patient are shown.

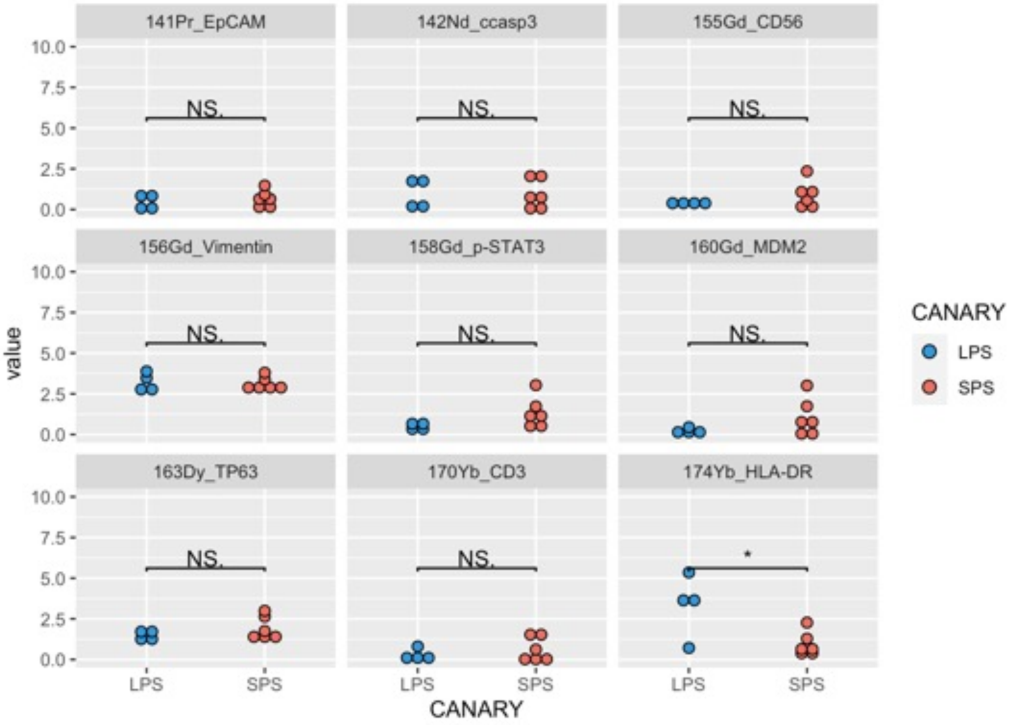

Figure E12. Protein expression comparison for fibroblasts/mesenchymal cells. Only the protein markers which have an average protein expression > 1.4 for at least one patient are shown.

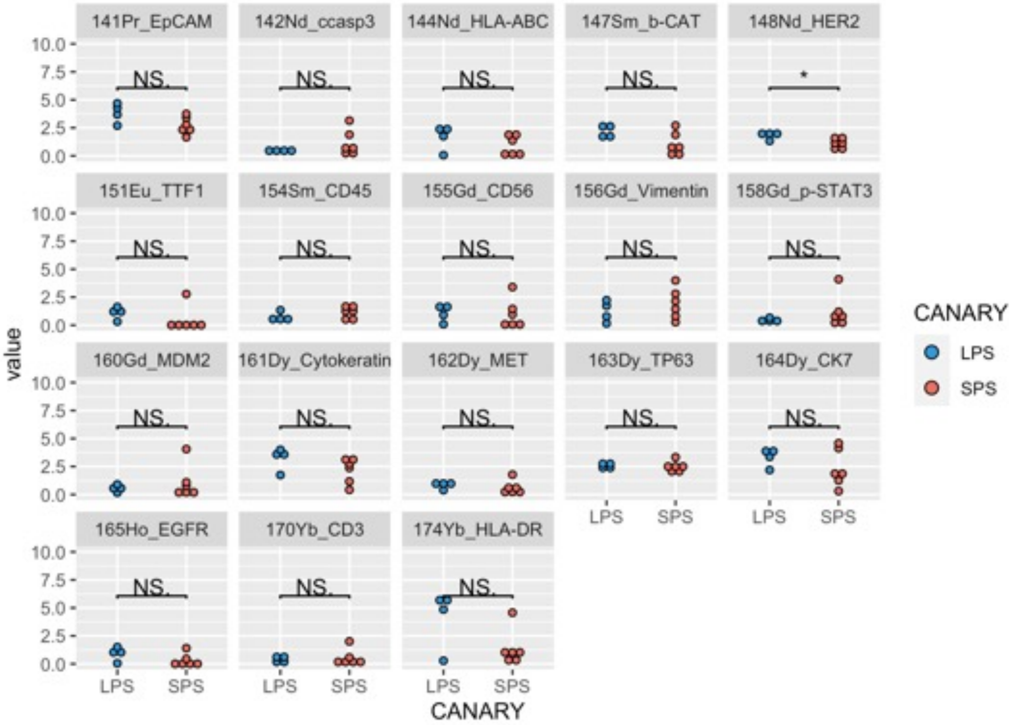

Figure E13. Protein expression comparison for epithelial cells. Only the protein markers which have an average protein expression > 1.4 for at least one patient are shown.

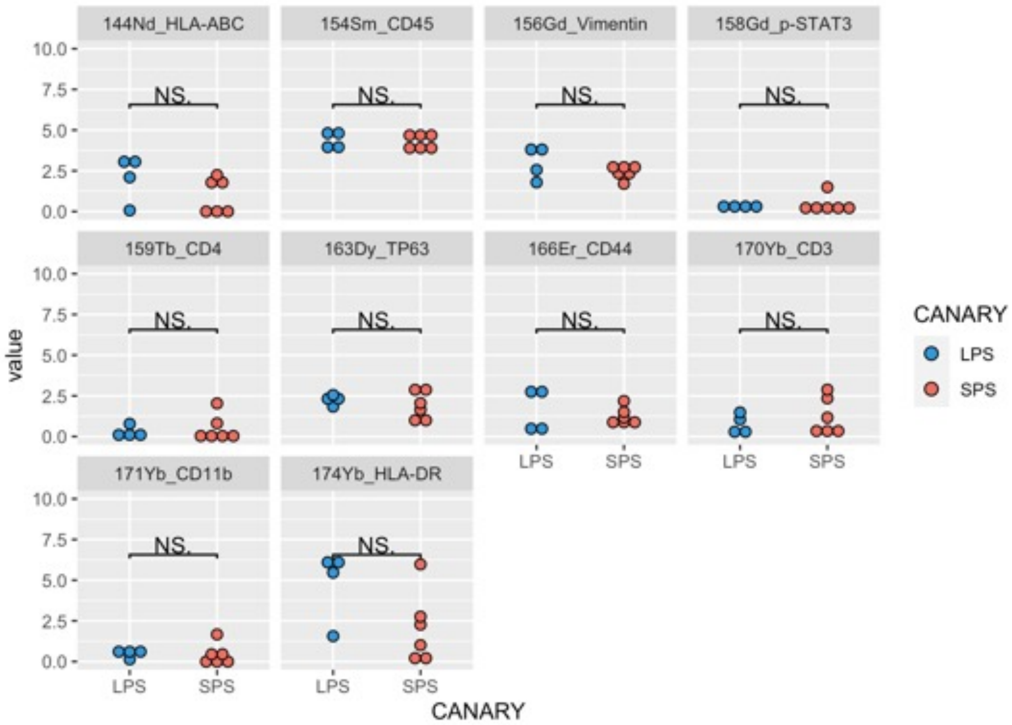

Figure E14. Protein expression comparison for immune cells. Only the protein markers which have an average protein expression > 1.4 for at least one patient are shown.

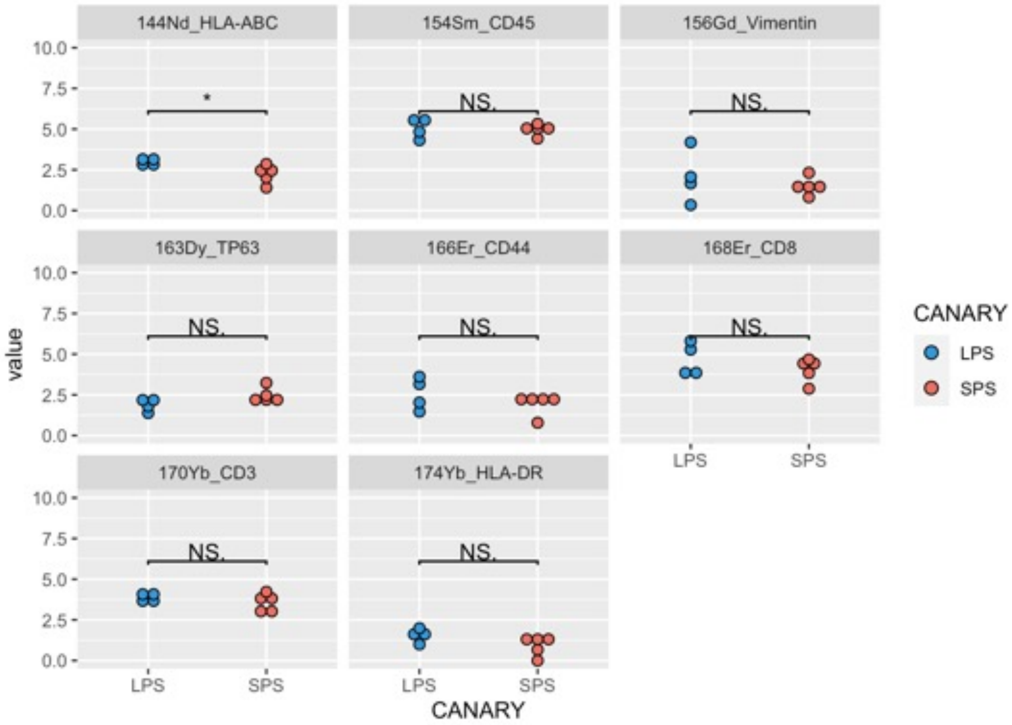

Figure E15. Protein expression comparison for CD8+ T cells. Only the protein markers which have an average protein expression > 1.4 for at least one patient are shown.

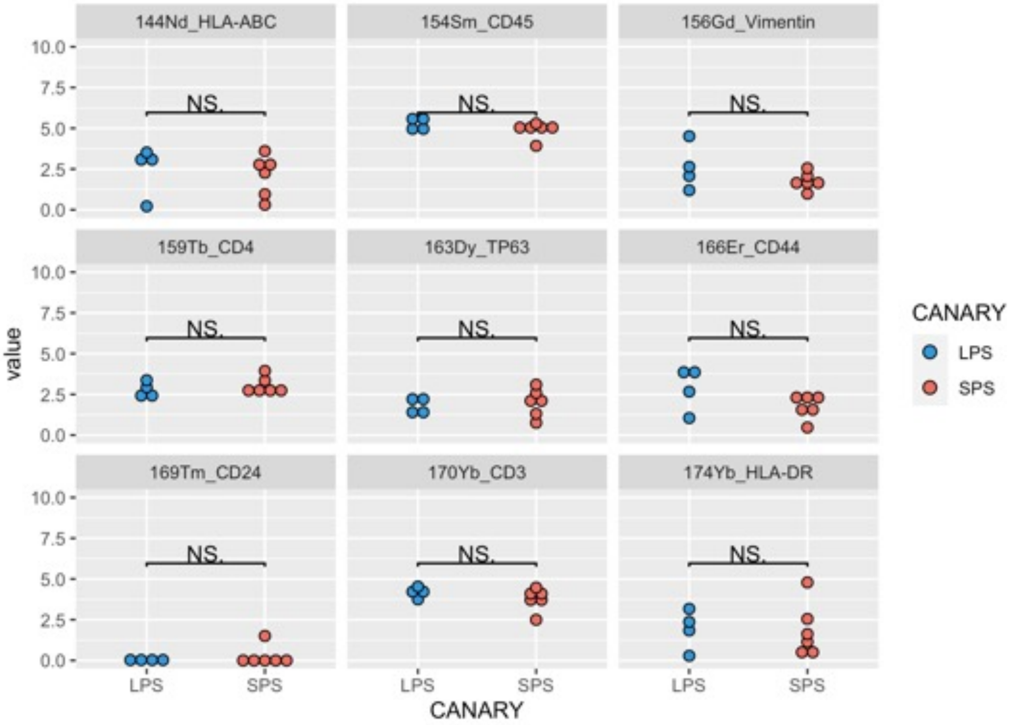

Figure E16. Protein expression comparison for CD4+ T cells. Only the protein markers which have an average protein expression > 1.4 for at least one patient are shown.

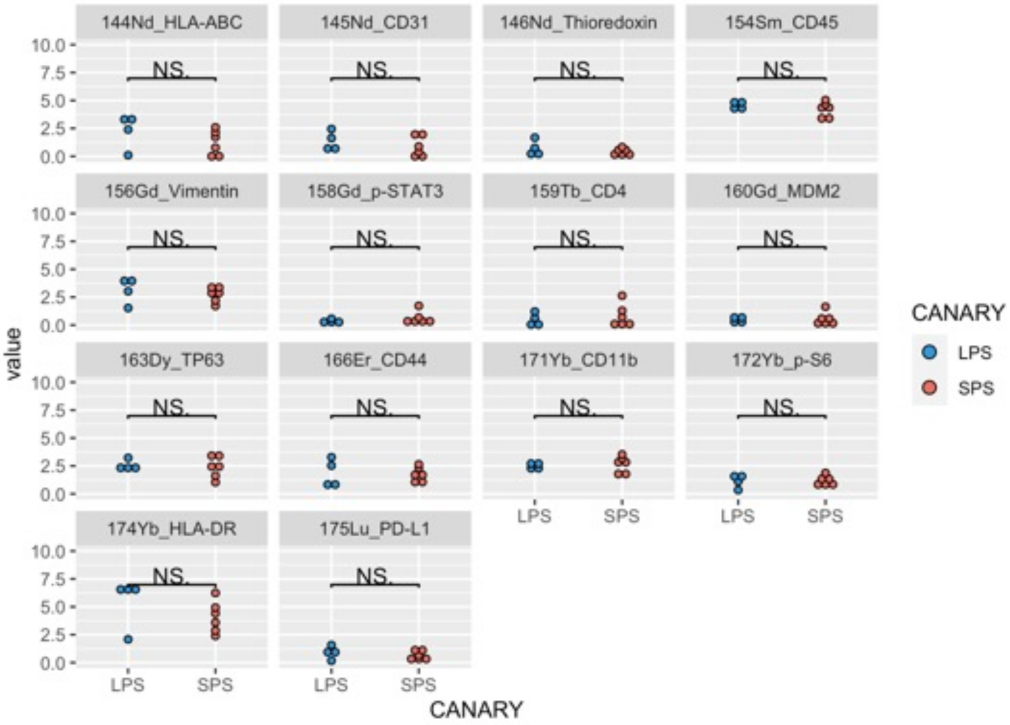

Figure E17. Protein expression comparison for myeloid cells. Only the protein markers which have an average protein expression > 1.4 for at least one patient are shown.

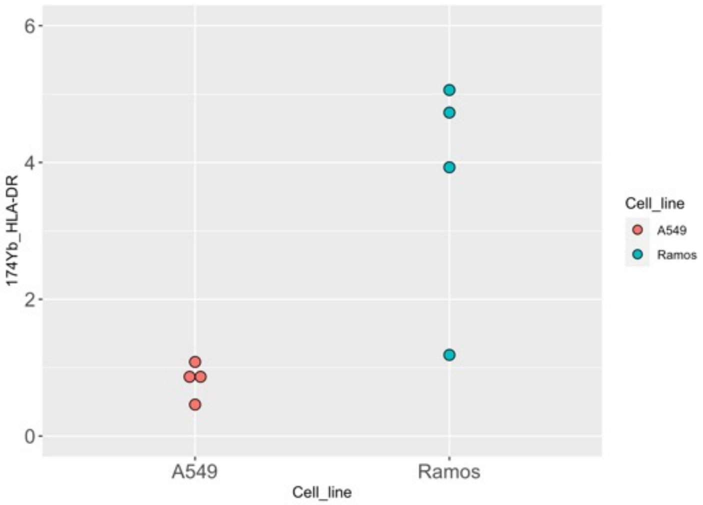

	Min	1st Qu.	Median	Mean	3rd Qu	Max
A549	0.4604	0.7593	0.8666	0.8193	0.9267	1.0836
Ramos	1.186	3.244	4.329	3.726	4.812	5.059

Figure E18. HLA-DR expression in batch control cell lines A549 and Ramos.

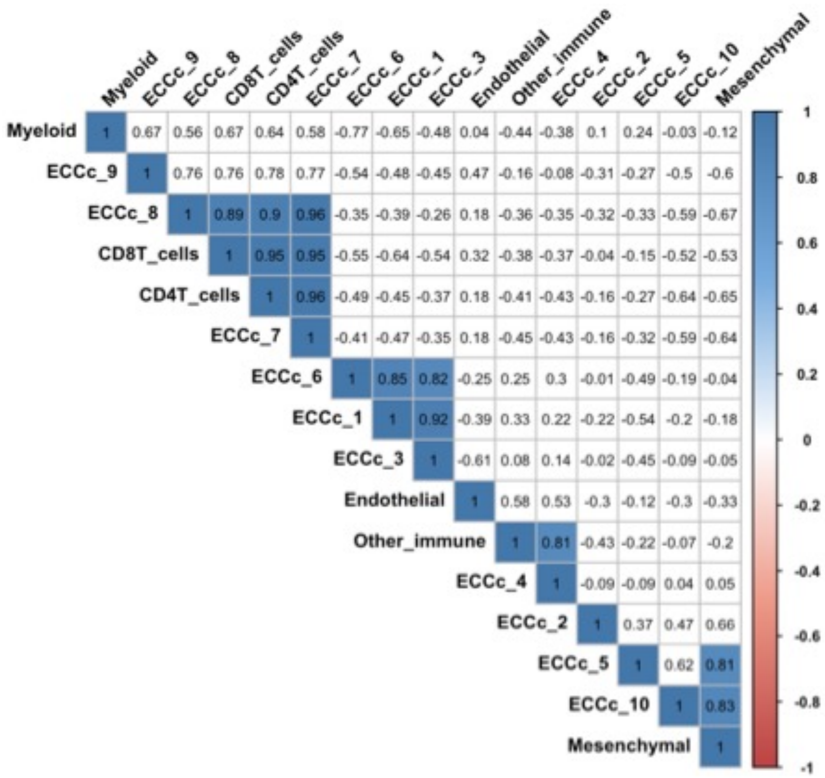

Figure E19. Spearman correlation of main all cell types and 10 epithelial clusters. Only significant correlations (p value >0.05) are colored. P values are adjusted for multiple hypothesis testing by Benjamini-Hochberg procedure.

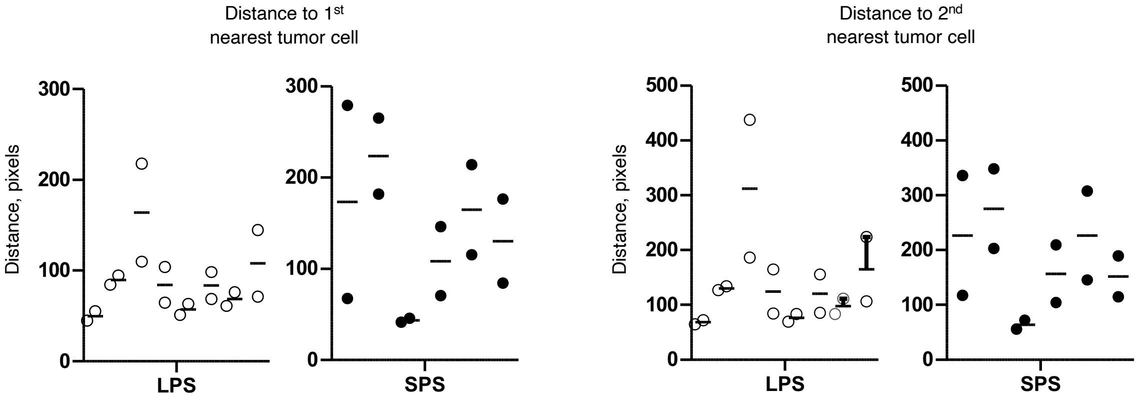

Figure E20. Extended Figure 5C showing results for each individual patient (2 cores/patient).
